# Temporal displacement of the mammal community in a protected area due to hunting and recreational activities

**DOI:** 10.1002/eap.70118

**Published:** 2025-11-02

**Authors:** Anne Peters, Adam F. Smith, Maik Henrich, Carsten F. Dormann, Marco Heurich

**Affiliations:** ^1^ Department of Wildlife Ecology and Management, Faculty of Environment and Natural Resources University of Freiburg Freiburg Germany; ^2^ Department of National Park Monitoring and Animal Management Bavarian Forest National Park Grafenau Germany; ^3^ The Frankfurt Zoological Society Frankfurt Germany; ^4^ Department of Biometry and Environmental System Analysis, Faculty of Environment and Natural Resources University of Freiburg Freiburg Germany; ^5^ Institute of Forestry and Wildlife Management, Inland Norway University of Applied Science Koppang Norway

**Keywords:** activity pattern, camera trap, conservation, hunting, nocturnality, protected area, recreation, tourism, wildlife management

## Abstract

Recreation (i.e., hiking and biking) and hunting can occur simultaneously in time and space, and both sources of disturbance affect wildlife behavior, leading to reactions resembling anti‐predator behavior. However, the additive effects of lethal and non‐lethal human disturbances on wildlife are only beginning to be understood, and research on the impact of hunting on non‐target species is limited. Recreation and hunting commonly co‐occur in areas where wildlife is present, and understanding their combined effects on wildlife behavior is crucial for protected area management. Using records from 122 camera traps placed along trails and in surrounding forests, we assessed the effect of varying intensities of hunting and recreation over space and time on the temporal activity of red deer (*Cervus elaphus*), roe deer (*Capreolus capreolus*), wild boar (*Sus scrofa*), red fox (*Vulpes vulpes*), and Eurasian lynx (*Lynx lynx*) in the Bavarian Forest National Park, Germany. We documented the relative abundance of these species on trails versus in forests and applied Bayesian models to assess how hunting and recreation influenced wildlife nocturnality. Our results suggest that hunting is a strong driver behind wildlife temporal behavior. Hunting amplified avoidance of non‐lethal recreation and potentially impacts species interactions. Red deer exhibited the most pronounced temporal avoidance of both hunting and recreational activity, increasing nocturnality and trail avoidance as these disturbances increased. Red deer were more diurnal in the non‐hunting zone and decreased nocturnal activity with increasing distance from the hunting zone. Wild boar and non‐hunted species exhibited moderate or negligible responses. However, high hunting effort led to species not targeted by hunting (roe deer and red fox) increasing their temporal avoidance of recreational activities, with wild boar and roe deer avoiding trails more strongly. In the context of protected area management, our results suggest that strictly reducing hunting in space and time while concentrating recreation in certain areas to create disturbance‐free habitat year‐round has great potential to reduce the temporal avoidance of humans by wildlife, thereby fostering nature conservation goals by protecting natural processes.

## INTRODUCTION

Human activity continues to increase globally, leading to habitat degradation and fragmentation, the primary causes of biodiversity loss (Caro et al., 2022; Otto, 2018; Tilman et al., 2017). Approximately 75% of the terrestrial environment has been strongly modified by human activities (Meng et al., 2021). Anthropogenic pressures have been found to drive the selection of traits toward those favoring survival in human‐modified areas (Ciuti et al., 2012; Otto, 2018). Alterations in wildlife behavior, that is, modifications of spatial and/or temporal activity, may translate into changes in intra‐ and interspecies interactions, potentially leading to cascading effects in the ecosystem (Gaynor et al., 2018; Tucker et al., 2018; Wilson et al., 2020). For instance, human activity may force prey to temporally overlap more with predators, potentially reducing prey survival rates (Bonnot et al., 2020; Gehr et al., 2018). Behavioral changes in ungulates may alter seed dispersal, grazing, and browsing, reshaping plant communities over time (Cherry et al., 2016; Wright et al., 2020). However, behavioral adjustments may benefit human–wildlife coexistence by reducing negative encounters (Carter et al., 2012; Gehr et al., 2017; Smith et al., 2024).

Alterations in wildlife behavior as a response to human activity occur because wildlife developed anti‐predator behaviors toward generally threatening stimuli (e.g., fast‐moving objects or loud noises). Moreover, humans have been lethal predators for all wildlife species for millennia (Frid & Dill, 2002). Hence, stimuli connected to human activity can be seen as analogous to predation risk, as stated by the risk‐disturbance hypothesis (Frid & Dill, 2002). Changes in behavior are driven by the risk that wildlife perceives from human activities in time and space. Behavioral adjustments are expected to be more pronounced when the perceived risk is higher (Frid & Dill, 2002), for instance in areas with higher human disturbance (Rogala et al., 2011; Suraci et al., 2019). Wildlife learns to adjust its behavior if the risk is spatially and temporally predictable (Creel et al., 2005; Gaynor et al., 2019). For example, wildlife may become more nocturnal in areas used by humans during the day to reduce encounters (Marion et al., 2021; Smith et al., 2024).

The risk‐disturbance hypothesis also applies to hunting‐related disturbances, as hunting effort impacts the risk wildlife perceives from human activities in time and space. Hunting can lead to human‐induced selection of certain behavioral traits (Ciuti et al., 2012; Otto, 2018), and animals can learn to adjust their behavior to different hunting tactics to increase survival (Thurfjell et al., 2013, 2017). Reducing daytime movement while increasing movement at night can be a response to avoid human hunters (Little et al., 2016; Ordiz et al., 2012; Stillfried et al., 2015). Moreover, both ungulates (Lone et al., 2015; Thurfjell et al., 2013; Zong et al., 2023a, 2023b) and carnivores (Brown et al., 2023; Ordiz et al., 2011) may use more concealed habitats to evade hunting disturbance. Overall, behavioral adjustments increase with hunting effort (Little et al., 2016; Scillitani et al., 2010; Stillfried et al., 2015). In areas with prolonged hunting effort, wildlife primarily avoids humans spatially, while temporal avoidance is more common during shorter hunting seasons (Parsons et al., 2022). Hence, the presence and intensity of hunting, both spatial and temporal, may impact the perceived threat of human presence for wildlife (Cromsigt et al., 2013; Lima & Bednekoff, 1999).

As a result, varying hunting effort can cause differences in wildlife responses to recreation, that is, non‐motorized activities such as hiking and biking. Recreational activities are a popular way for humans to use their natural environments, and protected areas are attractive destinations for many outdoor enthusiasts seeking to experience pristine nature (Balmford et al., 2015). However, protected areas play an increasingly important role in preserving vulnerable species and ecosystems (Meng et al., 2021), and increased human activity may impede nature protection. Recreational activities adversely affect wildlife behavior and physiology, triggering responses that resemble anti‐predator behavior, such as increased vigilance (Jayakody et al., 2008), flight behavior (Wam et al., 2014; Westekemper et al., 2018), altered movement patterns (Neumann et al., 2011; Ordiz et al., 2019), and physiological responses such as increased stress levels and heart rates (Reimoser, 2012; Zwijacz‐Kozica et al., 2013). In the long term, human disturbances can lead to changes in space use and temporal activity patterns (Gaynor et al., 2018; Heinemeyer et al., 2019). As recreational activities occur mostly during the day, wildlife becomes more nocturnal to avoid temporal overlap with humans (Marion et al., 2021). Trails are commonly used for recreation, hence wildlife associates trails with human disturbance and avoids them both temporally and spatially (Marion et al., 2021; Miller et al., 2017; Rogala et al., 2011). Higher trail use by recreationists intensifies this avoidance behavior (Muhly et al., 2011; Rogala et al., 2011). In contrast, denser vegetation cover can mitigate the impact of human activity on wildlife behavior by providing refuge habitat and reducing the perceived disturbance (Jayakody et al., 2008; Olson et al., 2018; Versluijs et al., 2022).

Recreation often occurs in the same area as hunting or in neighboring areas, making it more difficult for wildlife to correctly assess the risks that humans pose to them. Although wildlife can become habituated to recreational activities (Courbin et al., 2022; Reimers et al., 2010; Schuttler et al., 2017), most studies have not observed this effect, which may depend on the hunting effort wildlife is confronted with (Larson et al., 2016; Montgomery et al., 2020). Wildlife should overestimate rather than underestimate a risk to increase their chances of survival (Frid & Dill, 2002). Thus, we may reason that wildlife inhabiting areas in which hunting occurs perceive human presence as a threat due to negative experiences related to hunting and react toward recreational activities in ways resembling anti‐predator responses. However, research assessing the combined effect of hunting and non‐lethal recreation on wildlife behavior is scarce (Brown et al., 2023; Courbin et al., 2022; Kays et al., 2017; Marchand et al., 2014; Mols et al., 2022; Thiel et al., 2007). Disentangling the effects of single and combined disturbance sources is, however, important for a better understanding of their impact on wildlife behavior.

European national parks provide a unique opportunity to study the effects of varying intensities of hunting and recreation, as wildlife in many of these areas faces disturbances from both activities (Van Beeck Calkoen et al., 2020). This is because, in addition to conservation goals, the primary objective of national parks is to promote environmental education and recreation. At the same time, hunting is often practiced, at least along park borders, to reduce conflict with neighboring farmers or foresters due to browsing and crop damage (Ehrhart et al., 2016; Van Beeck Calkoen et al., 2020). Especially, the clear spatial division of hunting and hunting‐free zones in national parks provides an opportunity to assess the additive effects of hunting and recreation on wildlife.

We deployed camera traps in the Bavarian Forest National Park (BFNP) to monitor wildlife activity on trails and in nearby forests. Cameras placed on trails additionally recorded recreational activity. Hunting effort over time was determined using detailed data on successful hunting events inside the national park's hunting zone, as we assume those to increase with increasing hunting attempts. This setup provided the opportunity to determine how different intensities of recreation and hunting affect the temporal activity of wildlife. Five species were included in this study, representing species from all guilds of the large mammal community: (1) red deer (*Cervus elaphus*) and (2) wild boar (*Sus scrofa*), both group‐living ungulates which are currently hunted in the BFNP's hunting zone; (3) roe deer (*Capreolus capreolus*), a solitary herbivore that has not been subjected to hunting inside the BFNP since 2012; (4) red fox (*Vulpes vulpes*), a solitary mesocarnivore; and (5) Eurasian lynx (*Lynx lynx*), a solitary large carnivore, neither of which are subjected to hunting in the BFNP.

Red and roe deer typically show activity peaks at dusk and dawn year‐round (Ensing et al., 2014; Marion et al., 2021). Red deer remain mostly active during twilight but may increase daytime activity in winter due to abiotic factors, variations in human disturbance, and predator presence (Ensing et al., 2014; Kamler, 2007). Similarly, roe deer are more day‐active in winter but increase nocturnal and twilight activity in summer and autumn, driven by physiological and environmental factors (Krop‐Benesch et al., 2023; Pagon et al., 2013). Both species shift to diurnal or cathemeral behavior in areas with reduced recreation or hunting disturbance (Bonnot et al., 2020; Kamler, 2007; Marion et al., 2021). Wild boars are typically nocturnal (Johann et al., 2020; Reinke et al., 2021) but may increase diurnal activity in undisturbed areas during summer (Keuling et al., 2008; Reinke et al., 2021) or with low hunting and recreation pressure (De Assis Morais et al., 2020; Johann et al., 2020). Red foxes are nocturnal in disturbed areas (Díaz‐Ruiz et al., 2015; Kämmerle et al., 2020) but cathemeral in low‐disturbance areas (Ikeda et al., 2016). They may be slightly more diurnal during the breeding season (February–April; Monterroso et al., 2014) and in winter (Kämmerle et al., 2020), although Ikeda et al. (2016) reported less diurnal activity in winter. Eurasian lynxes are primarily nocturnal with activity peaks at twilight, unaffected by human disturbance (Podolski et al., 2013; Smith et al., 2024). This behavior likely supports their ambush‐hunting strategy in low‐light conditions (Smith et al., 2024).

**FIGURE 1:**
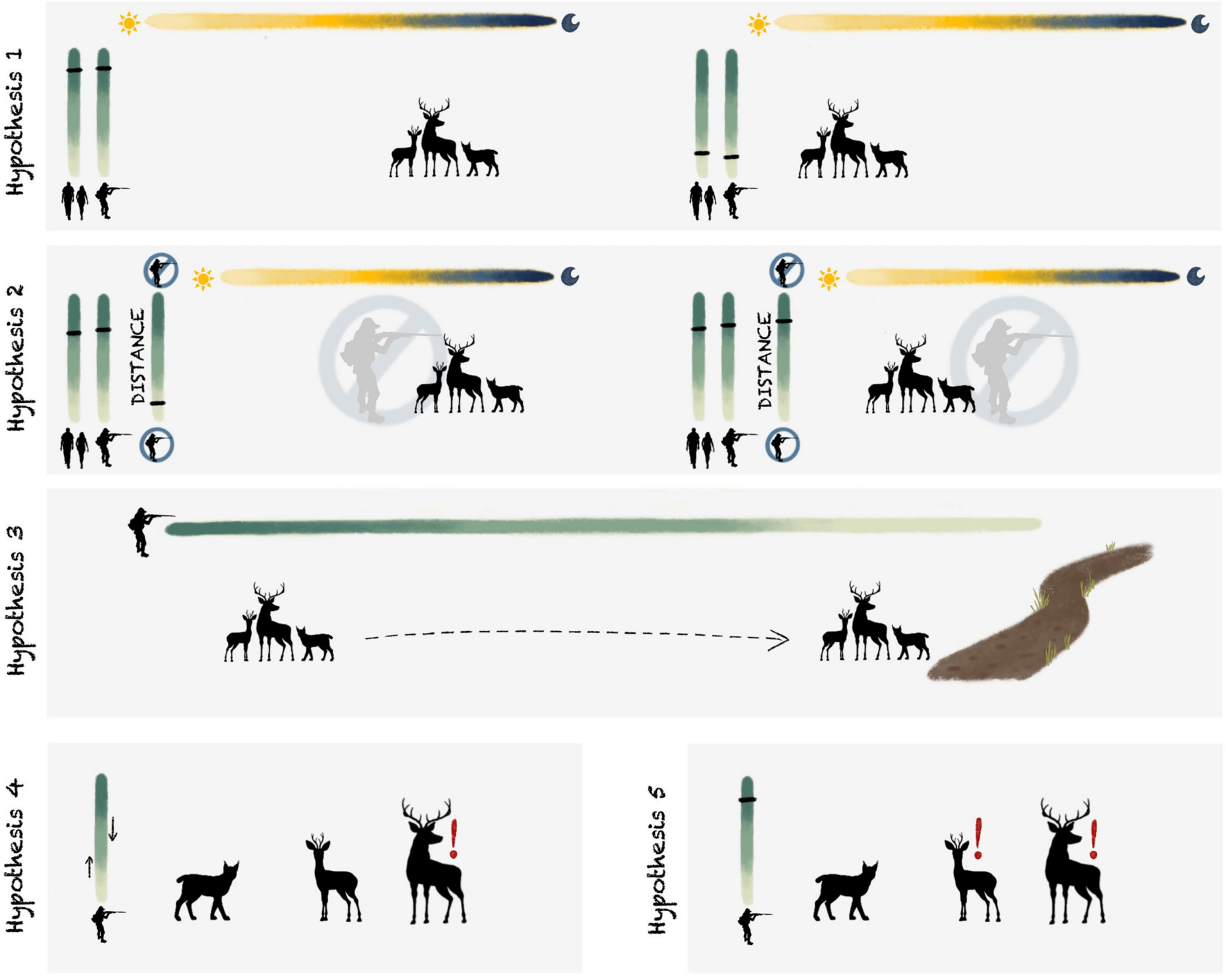
Graphical representation of the predicted behavioral responses of the target species toward recreation and hunting disturbances according to our hypotheses: (H1) the target species should be more nocturnal when hunting effort in the hunting zone and recreation are high but become more diurnal when these disturbances decrease; (H2) this effect would also be noticeable in the non‐hunting zone, but would weaken with increasing distance from the hunting zone; (H3) the target species will spatially avoid trails less as hunting effort decreases; (H4) changes in temporal activity and trail avoidance as a response to varying hunting effort would be more noticeable in hunted species (red deer and wild boar, represented by the red deer silhouette); (H5) non‐lethal disturbances from high hunting efforts could also affect non‐hunted species that experience hunting outside the Bavarian Forest National Park (roe deer and red fox, represented by the roe deer silhouette). Silhouettes, color scales and all other drawings by Anne Peters.

We hypothesized (H1) that the target species would adjust their temporal activity depending on the intensity from recreation/hunting disturbance (Figure 1). Specifically, we expect an increase in nocturnality as recreational activity on trails and/or hunting effort in the hunting zone increases. However, they should become more diurnal when recreational activity on trails and/or hunting effort in the hunting zone decreases. We further hypothesized (H2) that this effect would be noticeable even in the non‐hunting zone but would weaken with increasing distance from the hunting zone, that is, the area with the highest potential disturbance intensity. That is because less pronounced reactions are expected farther from anthropogenic disturbances. Considering spatial behavior, we hypothesized that (H3) the target species would exacerbate avoidance of infrastructure related to recreation activities, that is, trails, when hunting effort increases. Overall, we predicted ungulates to be more frequently recorded at forest locations. Nonetheless, we expected some ungulate presence on trails, for example, when crossing or feeding close to trails, which should, however, decrease with increasing hunting effort. Carnivores were expected to be more frequently recorded on trails overall, but less so with increasing hunting effort. We hypothesized that (H4) these changes in temporal activity and trail avoidance would be more noticeable in hunted species, that is, red deer and wild boar. These species regularly face human hunters and should be more cautious when hunting intensifies to increase their survival. In contrast, species not hunted for several years (roe deer, red fox) or never hunted (lynx) would be less reactive. However, we hypothesized that (H5) non‐lethal disturbances from high hunting efforts, such as noise from firearms, could also affect non‐hunted species. We predict that species once hunted and still hunted outside the national park, that is, roe deer and red fox, would be more sensitive to hunting disturbances. Lynx, which are protected, would be less sensitive to hunting disturbances due to a lack of hunting history in the national park and surrounding areas, except illegal killings (Heurich et al., 2018).

## METHODS

### Study area

**FIGURE 2:**
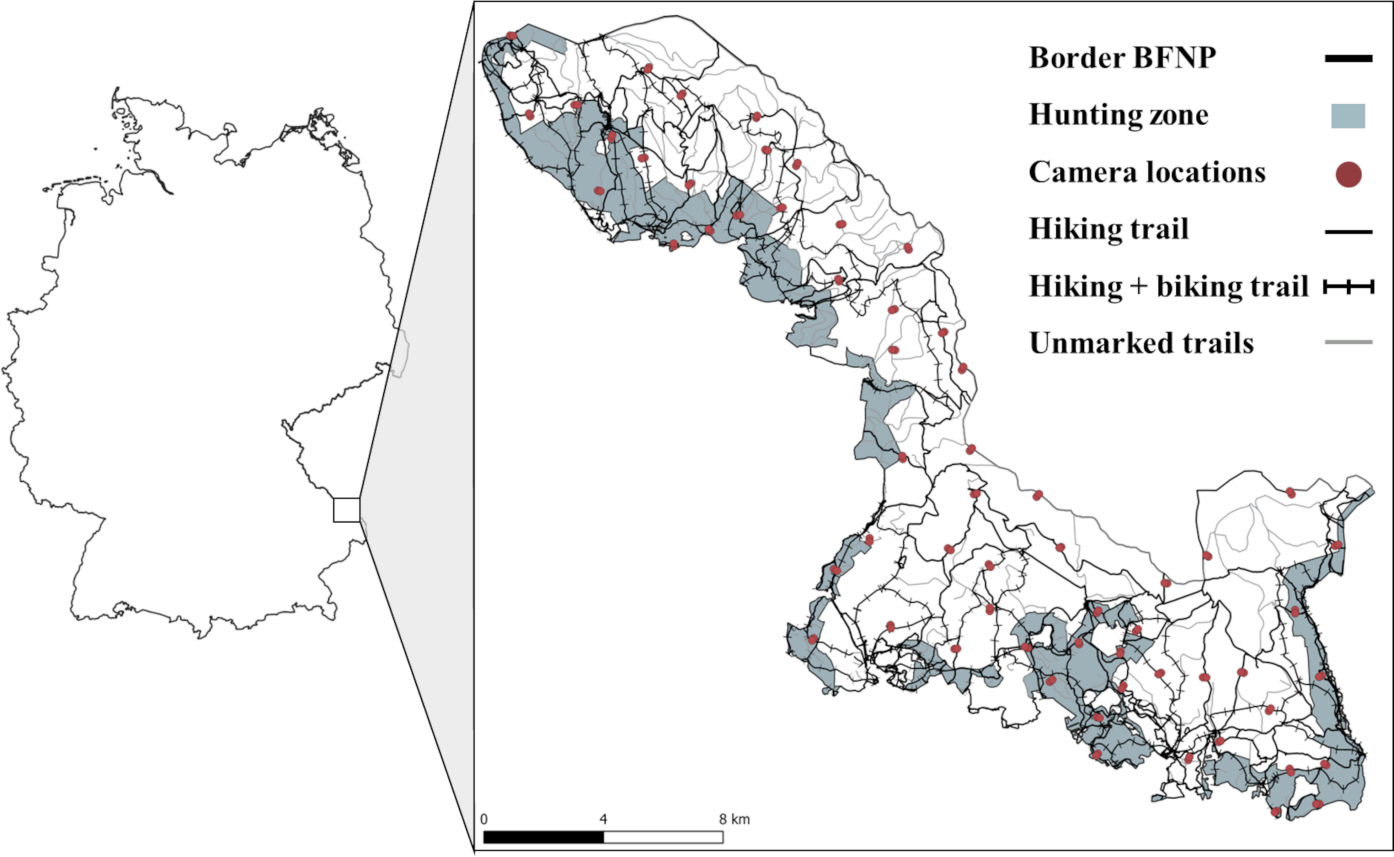
Map of the Bavarian Forest National Park (BFNP). Blue areas indicate the hunting zone and dark red dots represent camera trap locations. Solid black lines are hiking trails, solid black lines with vertical lines are multiuse trails (i.e., hike and bike) and gray lines are unmarked trails with temporally restricted access.

The BFNP was established in 1970 and is located along the Czech‐German border in south‐eastern Germany, covering an area of approximately 250 km^2^ (Figure 2). The elevation ranges from 600 to 1453 m asl, with subalpine spruce forests of Norway spruce (*Picea abies*) interspersed with mountain ash (*Sorbus aucuparia*) being the predominant forest type above 1100 m asl (Van Der Knaap et al., 2020). Mixed montane forests are the most common forest type, with Norway spruce, white fir (*Albies alba*), European beech (*Fagus sylvatica*), and Sycamore maple (*Acer pseudoplatanus*; Van Der Knaap et al., 2020), at elevations between 600 and 1100 m asl.

Approximately 1.3 million visitors are attracted to the BFNP each year. Hiking is more common than biking and can be conducted on a trail network of approximately 513 km, of which 217 km are marked as biking trails. Additionally, 258 km of unmarked, smaller trails can be used by hikers during their visits to the national park (Figure 2).

Red deer and wild boar are the only wildlife species regulated by hunting in the national park's hunting zone, which is located along the park border (Figure 2). The hunting season for red deer lasts from June to January, whereas wild boar is hunted year‐round, both during day and night, and more intensively than red deer. Hunters are usually active during twilight (Appendix S1: Figure S1), and hunting practices include hunting from high seats, attracting wildlife with bait, and using enclosures (Möst et al., 2015) or boar traps to cull the animals.

### Camera trap placement and picture classification

A total of 122 camera traps were deployed from November 2020 to early December 2021 at 61 locations on trails and 61 off‐trail locations in the forest. The camera trap models Cuddeback C2 and Cuddeback G were used, both of which were equipped with a black flash. Each camera was set to capture three consecutive images with minimal delay after being triggered.

To place cameras on trails, 60 random points were generated along the trail network based on a systematic random design using Quantum GIS 3.32.2 (QGIS Development Team, 2022). Cameras on trails were installed 50 cm above the trail surface and aligned at a 90° angle to the trail. Forest cameras were deployed at a 100 m distance and at a 90° angle to the on‐trail camera. Forest cameras were also placed at a lens height of 50 cm but were directed northward to avoid sunlight reflections in the image. Camera placement followed criteria outlined in Appendix S1: Table S1. If these criteria were not met due to unsuitable random placement of the on‐trail camera (e.g., too close to the forest edge so that the forest camera could not be placed in the same habitat), the on‐trail camera was manually relocated along the same trail until the criteria for both on‐trail and forest cameras were satisfied. During the study, one camera pair had to be repositioned, resulting in 61 locations overall, with 60 on‐trail and forest cameras simultaneously being active.

Captured images were classified using the open‐source detection model MegaDetector 4.0 (Beery et al., 2019) to filter out empty images and to determine images containing only humans and could, therefore, be reliably determined as hikers (Mitterwallner et al., 2023). To satisfy individual privacy regulations, bounding boxes around humans were blackened using Amazon Rekognition (Amazon Web Services, 2023), an artificial intelligence software. Images including object categories such as “vehicle” or “animal” were manually classified using the server‐based software TRAPPER (Bubnicki et al., 2016) to determine the type of human activity and the animal species. For instance, images containing the categories “human” and “vehicle” may depict either a person driving a car or a biker, which was not possible to differentiate from the AI classifications alone. All non‐motorized human activities on the trails, mainly hiking and biking, were used to determine human disturbance on the trails without differentiating between activities. Occasionally, national park employees were present on foot during their duties, but these were not excluded, as we assume that wildlife likely does not distinguish them from recreational visitors. Motorized human activities in the national park are rare and mainly consist of employees using cars or forestry machines and are therefore irrelevant to recreational activities.

## DATA ANALYSES

All analyses were conducted using the statistical software “R” (v4.3.0, R Core Team, 2023).

### Hunting effort

To assess how varying hunting effort affects the behavior of the target species, we used hunting data collected by national park game wardens from November 2020 to December 2021, assuming that a higher number of culled animals indicates a higher hunting effort. Based on the number of animals culled per month in relation to the respective year, the camera trap dataset was split into low (February–May, <5% of all hunting events), medium (June–September, 5%–10% of all hunting events), and high (October–January, >10% of all hunting events) hunting effort. Because camera traps were deployed from mid‐November 2020 to early December 2021, the “first” high hunting effort period encompasses November 2020 to January 2021, while the “second” high hunting effort period covers October to December 2021. To ensure sufficient data for analyses during high hunting effort, camera trap data from both high hunting effort periods were combined to increase the number of wildlife events available.

### Recreational activity on trails

To determine the intensity of recreational activities on trails and their impact on the target species, data from on‐trail cameras were used as humans are mostly active on trails and during the day. Recreationists usually pass by cameras quickly, spending little time in view, unlike wildlife, which may linger for several minutes, for example, while feeding. However, on heavily used trails, different groups of recreationists may follow in quick succession, triggering the same camera within a short time. To address this, an 8‐second separation interval was used to define independent events of recreational activities. A relative abundance index (RAI) for recreational activities was calculated for each on‐trail camera by dividing the number of independent events recorded by the total number of days the camera was active. This was done per camera and hunting effort to account for spatiotemporal variation in recreational activity. When calculating trapping days, days were excluded if the camera was inactive or obstructed, for example, due to snow covering the lens (see Appendix S1: Table S2 for details on trapping days). Resulting RAIs were multiplied by 100 as previously done by Henrich et al. (2022).

### Wildlife relative abundance and trail index

To analyze the effect of hunting effort on wildlife trail use, the RAI was calculated separately for on‐trail and forest cameras for each target species. In this way, we could determine if a species was observed more frequently on trails or in forests during periods of different hunting efforts. A 5‐minute separation interval was used to define independent wildlife events (Henrich et al., 2022). We did not count the number of individuals per event because group size was irrelevant to our analysis. For instance, a sounder of 10 wild boars should count the same as one wild boar when determining if the species was generally observed more often on trails than in forests. We derived the RAI by dividing the number of independent events recorded by all on‐trail or forest cameras by the total number of days those cameras were active, multiplied by 100 (Henrich et al., 2022). To calculate the 95% CI, we resampled the data at the level of camera trap locations 1000 times using nonparametric bootstrapping and recalculated the RAI over all camera locations.

Subsequently, we calculated a trail index based on Bitetti et al. (2014), however, using the RAIs instead of the number of events to correct for differences in trapping days across hunting efforts and camera traps. We calculated the trail index for all species based on Equation (1) for each hunting effort. A trail index below zero indicates that the species was more often recorded in forests, whereas an index above zero indicates that the species was recorded more frequently on trails. To estimate the trail index's 95% CI for each species, we resampled camera locations with replacement 1000 times using nonparametric bootstrapping.

(1) $$\;\text{Trail index}=\log\left(\frac{{\mathrm{RAI}}_{\text{trail}}+1}{{\mathrm{RAI}}_{\text{forest}}+1}\right)$$

For each target species, we calculated the differences in RAI estimates between on‐trail and forest cameras, along with the 95% CIs of these differences, to determine whether the RAI significantly varied between trail and forest locations during a specific hunting effort (following the same method as Henrich et al., 2024). A significant difference would be indicated if the CI did not overlap zero. Additionally, we tested whether the trail index differed significantly across hunting efforts (high vs. low, high vs. medium, and medium vs. low) by calculating the differences between the respective trail index estimates and their 95% CIs.

### Activity patterns

**FIGURE 3:**
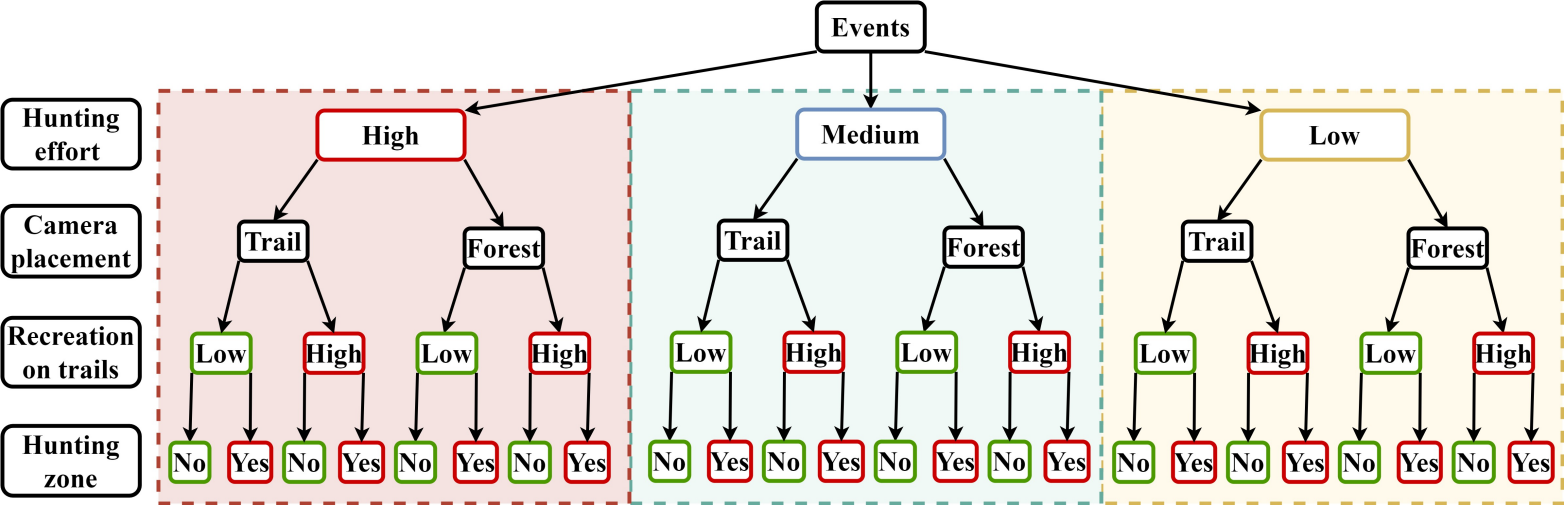
Schematic diagram of subsets generated to calculate activity curves. To display a human disturbance gradient, the datasets containing events of the target species were split into different subsets using hunting effort (high, medium, and low), camera placement (trail/forest), recreation activity on trails (low/high), and information on the camera being placed inside or outside of the hunting zone.

To examine how the activity patterns of the target species changed over a gradient of human disturbance intensity, we created 24 subsets of camera trap data for each target species, representing different intensities of recreational and hunting disturbances (Figure 3). The dataset was split by hunting effort and further divided into camera placement, that is, on‐trail or forest. Cameras were then grouped based on the RAI of recreational activity on the trail in camera locations with “low” and “high” recreational disturbance. We categorized recreational activity because activity curves cannot display continuous variables, but we were interested in whether wildlife would be less nocturnal if recreational activities were lower. Using the median RAI of recreational activities on trails for each hunting effort, the 30 cameras with a lower RAI were categorized as “low,” and the 30 cameras with an RAI higher than the median were defined as “high” recreational activity. This grouping was done separately for the two high hunting effort periods (November 2020 to January 2021 and October to December 2021) to capture differences in recreational activity at each camera location between the two years, but the periods were later combined to increase the number of available wildlife events. Likewise, the paired forest cameras were categorized into “high” and “low” recreational activity using information from the trail camera to determine the impact of recreational activity on nearby trails on the target species. Only two categories of recreation intensity (low and high) were used, as further subdivision resulted in many subsets with too few events of the target species to ensure robust activity pattern estimates. Finally, the dataset was divided into cameras placed in the hunting and non‐hunting zones (Figure 3).

Activity patterns were calculated for each subset using the R package “activity” (Rowcliffe, 2023). Rowcliffe et al. (2014) suggested that at least eight independent events should be used to estimate activity patterns. Based on this suggestion, we excluded subsets with fewer than 10 independent events of the target species. Wildlife and human activity periods were determined using solar time to account for daylight shifts between periods with different hunting efforts. Clock time was converted to solar time using the function “solartime” from the package “activity” (Rowcliffe, 2023). We used the function “fitact” from the package “activity” (Rowcliffe, 2023) to generate density curves of the target species' activity using 1000 bootstraps.

### Nocturnality model

We hypothesized that higher intensities of recreation and hunting effort and a closer proximity to these disturbances should lead to the target species becoming more nocturnal. To test our hypotheses, we fitted Bayesian generalized linear models using the function “brm” from the R package “brms” (Bürkner, 2021). The Bayesian approach was chosen to manage the complexity of three‐way interactions involving hunting effort, camera placement, and each of the other variables included in the model. Wildlife events were divided into those recorded during the day (end of morning nautical twilight to the onset of evening nautical twilight) and those recorded during the night (beginning of evening nautical twilight to the end of morning nautical twilight; Ensing et al., 2014) and served as response variables in a beta‐binomial model. This family was chosen to account for overdispersion of the data.

Hunting effort (low, medium, and high) and camera trap placement (trail or forest) were included as categorical covariates. To assess the effect of the non‐hunting zone, the distance of each camera to the border of the hunting zone was calculated using Quantum GIS 3.32.2 (QGIS Development Team, 2022), with a distance of zero meters indicating cameras located inside the hunting zone. Recreation intensity was included as the RAI of recreational activity on the trail for each camera pair for each period of differing hunting efforts, combining the two high hunting effort periods. To determine if vegetation cover functions as a potential buffer from human disturbances, we included the mean visibility around each camera in a 50 m buffer as a control variable. Mean visibility was calculated using Quantum GIS. Visibility maps were generated using terrestrial laser scanning to determine forest openness from 0 to 50, 70, and 140 cm above the ground, with 0 indicating low and 1 indicating high visibility. Using information from airborne laser scanning across the BFNP, a random forest model was used to extrapolate the visibility derived from terrestrial laser scanning across the study area (Zong et al., 2021, 2023a, 2023b). Different visibility layers were used to include the height of vegetation cover relevant to a particular species depending on the approximate eye level; for example, 140 cm for red deer and 50 cm for red foxes. To assess the effects of hunting effort and camera placement (trail/forest) on wildlife nocturnality, we included a three‐way interaction between hunting effort, camera placement, and the other variables (distance to the hunting zone, RAI of recreational activity, and visibility). Variables were always scaled using the base R function “scale,” whereas quadratic terms or log‐transformations were included when residuals were not uniformly distributed. Residual diagnostics were performed using the “DHARMa” package (Hartig, 2022). The variables included and the species‐specific formulas are detailed in Table 1, Appendix S1: Table S3.

**TABLE 1:** Name, range, median, and mean ± SD of the variables used in the models.

Variable name	Range	Median	Mean ± SD	Description/hypotheses
Hunting effort	High, medium, low			Displays varying hunting effort over the year. Higher hunting effort should increase nocturnality (Little et al., 2016; Parsons et al., 2022).
Camera placement	Trail, forest			Wildlife is expected to be more nocturnal on trails (Filla et al., 2017; Westekemper et al., 2018).
Distance to hunting zone (m)	0–3933	316	854 ± 1003	Increasing distance to the hunting zone should display less disturbance by hunting, thereby decreasing nocturnality.
RAI recreation on trails (high hunting)	23–6162	444	936 ± 1198	Displays levels of recreational activity on trails for each hunting effort. Higher levels of recreational activity should lead to increasing wildlife nocturnality to avoid humans (Gaynor et al., 2018).
RAI recreation on trails (medium hunting)	0–9124	784	1495 ± 2002	
RAI recreation on trails (low hunting)	5–4037	198	523 ± 729	
Visibility 140 cm	0.71–0.97	0.89	0.88 ± 0.062	Vegetation can provide cover, making wildlife less sensitive to disturbance in areas with higher coverage (Belotti et al., 2018; Stankowich, 2008) and hence decrease nocturnality. Expected to be mainly important for medium hunting effort (June–September) when vegetation has leaves.
Visibility 70 cm	0.67–0.96	0.89	0.88 ± 0.065	
Visibility 50 cm	0.38–0.65	0.51	0.51 ± 0.068	

*Note*: We included variables that represent different types of human disturbances (hunting effort, distance to hunting zone, and RAI recreation on trails) or may affect the disturbance intensity wildlife perceives from human activities (camera placement and visibility).

Abbreviation: RAI, relative abundance index.

## RESULTS

### Relative abundance and trail index

Red deer was the most frequently recorded ungulate species, followed by roe deer and wild boar (Table 2). For red deer, there was no significant difference in RAI between forest and trail cameras (Tables 3 and 4, Appendix S1: Figure S2). Roe deer had a significantly higher RAI on forest cameras than on‐trail cameras during medium hunting effort. Similarly, wild boar had a significantly higher RAI on forest cameras during both high and medium hunting efforts. The RAI of lynx and red fox was significantly higher on cameras placed on trails compared to in forests across all hunting efforts.

**TABLE 2:** Number of independent events of the target species based on a 5‐min independence threshold recorded by cameras placed on trails (sum trail) and in the forest (sum forest) as well as the mean number of independent events recorded by cameras on trails (mean trail) and in forests (mean forest) during high, medium, and low hunting effort.

Hunting effort	No. events	Red deer	Wild boar	Roe deer	Lynx	Red fox
High	Sum trail	394	82	115	127	304
	Mean trail	6.46	1.34	1.89	2.08	4.98
	Sum forest	459	241	208	10	44
	Mean forest	7.78	4.08	3.53	0.17	0.75
Medium	Sum trail	567	192	193	64	403
	Mean trail	9.30	3.15	3.16	1.05	6.61
	Sum forest	582	329	497	7	37
	Mean forest	9.86	5.58	8.42	0.12	0.63
Low	Sum trail	405	171	201	75	279
	Mean trail	6.64	2.80	3.30	1.23	4.57
	Sum forest	285	265	207	9	50
	Mean forest	4.83	4.49	3.51	0.15	0.85

**TABLE 3:** Relative abundance index (RAI) and trail index including lower and upper confidence intervals (CI) at different hunting efforts (high, medium, and low) for the target species on cameras located on trails (RAI trail) and in the forest (RAI forest).

Hunting effort/RAI/trail index	Red deer	Wild boar	Roe deer	Lynx	Red fox
High hunting					
RAI trail Lower/upper CI	5.55 3.82/7.65	1.15 0.82/1.57	1.62 0.61/3.37	1.79 1.31/2.36	4.28 2.95/6.01
RAI forest Lower/upper CI	7.03 5.09/10.01	3.73 2.42/5.38	3.14 1.65/5.38	0.15 0.08/0.25	0.66 0.31/1.25
Trail index Lower/upper CI	−0.20 −0.65/0.20	−0.79 −1.15/−0.42	−0.46 −1.12/0.21	0.88 0.69/1.08	1.15 0.77/1.55
Medium hunting					
RAI trail Lower/upper CI	7.88 5.37/10.91	2.77 1.97/3.76	2.58 1.78/3.56	0.92 0.56/1.33	5.77 4.22/7.64
RAI forest Lower/upper CI	8.54 5.91/11.36	7.08 5.15/9.76	4.86 3.33/6.70	0.10 0.03/0.20	0.53 0.29/0.82
Trail index Lower/upper CI	−0.07 −0.35/0.39	−0.76 −1.13/−0.42	−0.49 −0.83/−0.04	0.56 0.34/0.76	1.49 1.21/1.82
Low hunting					
RAI trail Lower/upper CI	5.95 4.02/8.77	2.51 1.52/3.97	2.69 2.01/4.84	1.10 0.78/1.57	4.10 3.06/5.45
RAI forest Lower/upper CI	4.50 3.14/6.08	4.09 2.67/5.96	3.29 2.01/4.78	0.14 0.06/0.29	0.79 0.50/1.18
Trail index Lower/upper CI	0.24 −0.26/0.40	−0.37 −0.85/0.16	−0.08 −0.50/0.37	0.61 0.41/0.81	1.05 0.76/1.36

*Note*: The trail index displays the relation between a species occurring on trails and in the forest. A trail index above 0 indicates that a species was more frequently recorded on cameras located on trails, while a trail index below 0 shows that a species was more frequently recorded in forests. A trail index of zero would indicate equal numbers of observations between trail and forest locations. CIs (lower/upper CI) were computed using nonparametric bootstrapping for both the RAI and trail index.

**TABLE 4:** The upper section of the table presents the difference in sample estimates (difference) and the corresponding lower and upper CIs (lower/upper CI) for comparisons of bootstrapped relative abundance index (RAI) values between trail and forest cameras under different hunting efforts (high, medium, and low).

Hunting effort	Difference in sample estimates	Red deer	Wild boar	Roe deer	Lynx	Red fox
RAI trail versus forest						
High	Difference Lower/upper CI	1.49 –1.40/4.68	2.57 **1.18/4.27**	1.52 −0.61/3.94	−1.64 **−2.21/−1.13**	−3.62 **−5.45/−2.19**
Medium	Difference Lower/upper CI	0.66 −3.06/4.45	2.28 **2.18/7.11**	2.28 **0.54/4.31**	−0.82 **−1.23/−0.45**	−5.25 −**7.11/−3.65**
Low	Difference Lower/upper CI	−1.46 −4.45/1.03	1.58 −0.40/3.81	0.34 −1.63/2.1	−0.96 **−1.42/−0.61**	−3.31 **−4.73/−2.22**
Trail index						
High versus medium	Difference Lower/upper CI	−0.13 −0.69/0.42	−0.02 −0.50/0.52	0.04 −0.75/0.77	0.33 **0.06/0.59**	−0.33 −0.85/0.14
High versus low	Difference Lower/upper CI	−0.44 **−0.71/−0.11**	−0.41 −0.94/0.06	−0.38 −0.82/−0.02	0.27 **0.04/0.52**	0.11 −0.35/0.52
Medium versus low	Difference Lower/upper CI	−0.31 −0.84/0.3	−0.39 −0.97/0.17	−0.41 −0.97/0.14	−0.06 −0.33/0.23	0.44 **0.02/0.86**

*Note*: Significant differences in RAI between forest and trail locations are indicated by CIs that do not overlap zero and are shown in bold. The lower section of the table shows the difference in sample estimates (Difference) and lower/upper CIs for comparisons of trail indices (which indicate a species' relative occurrence on trails versus in the forest, with positive values reflecting higher occurrence on trails) across hunting efforts (high vs. medium, high vs. low, and medium vs. low). CIs not overlapping zero indicate a significant difference in trail indices between the compared hunting efforts and are shown in bold.

The trail index further highlighted these patterns. During high and medium hunting efforts, all three ungulates were recorded more often on forest cameras than trail cameras, indicated by negative trail indices (Table 3, Appendix S1: Figure S2). During low hunting effort, the trail index increased for all three species, and red deer were recorded more often on trails than in forests (Tables 3 and 4, Appendix S1: Figure S2). Red deer showed a significant increase in the trail index during low compared to high hunting effort (Table 4, Appendix S1: Figure S2). Lynx and red fox consistently had positive trail indices, indicating a preference for trails across all hunting efforts (Table 3, Appendix S1: Figure S2). For lynx, the trail index increased significantly during high hunting effort compared to both medium and low hunting efforts (Table 4, Appendix S1: Figure S2). For red fox, the trail index increased significantly during medium hunting effort compared to low hunting effort (Table 4, Appendix S1: Figure S2).

### Activity patterns

**FIGURE 4:**
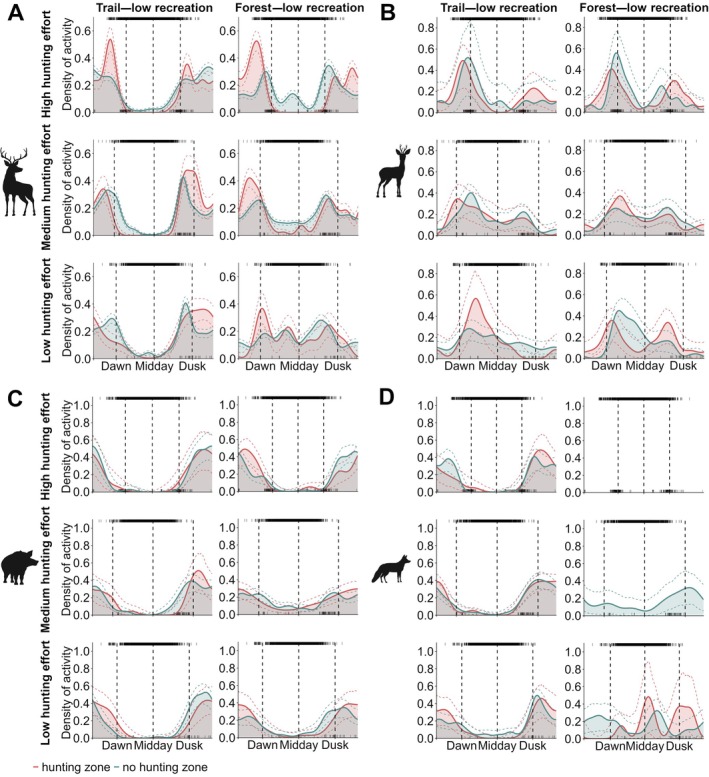
Activity density plots for red deer (A), roe deer (B), wild boar (C), and red fox (D) under different human disturbance intensities. The red curves represent activity density curves in the hunting zone, while the blue curves represent activity density curves in the non‐hunting zone. Dotted lines indicate CIs. Each subplot is organized by hunting effort, with high effort shown in the top row, medium in the middle row, and low in the bottom row. For each subplot, the left column shows results for cameras on trails with low recreational activity, and the right columns show results of forest cameras near trails with low recreational activity. The top rug in each plot shows the temporal distribution and density of recreational activity on trails, while the bottom rug shows the temporal distribution and density of successful hunting events. Dashed vertical lines mark dawn (left), midday (middle), and dusk (right), based on the sun's position. Missing curves indicate that there were not enough independent observations (no. observations < 10) of a certain species to produce reliable activity estimates. See Appendix S1: Figures S3–S6 for plots including results for trail and forest locations with high recreational activity. Animal silhouettes by Anne Peters.

Based on the activity curves, red deer were inactive on‐trail during the day, regardless of recreational activity intensity (Figure 4A, Appendix S1: Figure S3). However, their diurnal activity on trails slightly increased as hunting effort decreased. Red deer diurnal activity in forests increased with decreasing hunting effort, even when recreational activity on nearby trails was high. This trend was more pronounced in the non‐hunting zone. When recreational activity on trails was low, red deer consistently showed diurnal behavior on forest cameras in the non‐hunting zone. Under the same conditions in the hunting zone, red deer were nocturnal if hunting effort was high and became increasingly diurnal as hunting effort decreased, eventually exhibiting mostly diurnal activity in both the hunting and non‐hunting zones under low hunting effort.

Roe deer were predominantly crepuscular in the hunting zone, both on trails and in the forest under high hunting effort (Figure 4B, Appendix S1: Figure S4). In the non‐hunting zone, they showed increased daytime activity during high hunting effort but remained primarily crepuscular. Under medium and low hunting effort, roe deer were mostly diurnal, with peaks in the morning and afternoon, regardless of recreational activity intensity on trails or hunting zonation.

Wild boar were generally nocturnal on trails and in forests, showing little variation in their activity patterns across different intensities of hunting effort and recreational activity (Figure 4C, Appendix S1: Figure S5). However, in forests nearby trails with low recreational activity, wild boar exhibited slightly more daytime activity and less pronounced crepuscular peaks during medium hunting effort.

Red foxes were primarily nocturnal on trails but increased diurnal activity slightly as hunting effort decreased (Figure 4D, Appendix S1: Figure S6). Although less frequently recorded by forest cameras, the available data indicate that red foxes increased their activity during the day in forests under low hunting effort and were almost equally active during day and night.

**FIGURE 5:**
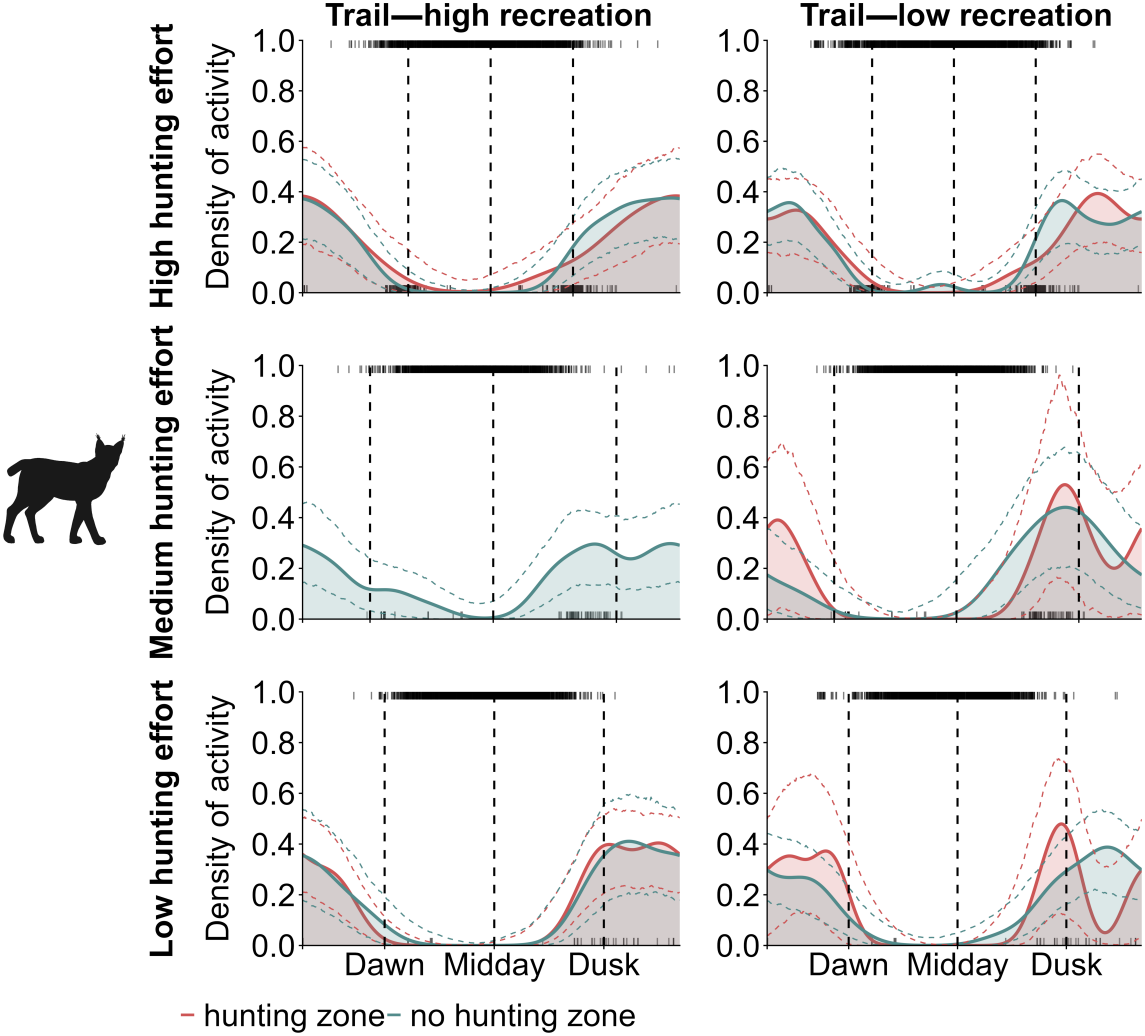
Activity density plots for lynx. The red curves represent activity density curves in the hunting zone, while the blue curves represent activity density curves in the non‐hunting zone. Dotted lines indicate CIs. The plots are organized by hunting effort, with high effort shown in the top row, medium in the middle row, and low in the bottom row. The columns show results for cameras on trails with high recreational activity (left column) and cameras on trails with low recreational activity (right column). The top rug in each plot shows the temporal distribution and density of recreational activity on trails, while the bottom rug shows the temporal distribution and density of successful hunting events. Dashed vertical lines mark dawn (left), midday (middle), and dusk (right), based on the sun's position. Missing curves indicate that there were not enough independent observations (no. observations < 10) to produce reliable activity estimates. Animal silhouettes by Anne Peters.

Since lynxes were rarely recorded on forest cameras, no activity plots were generated for forest locations. On trails, lynxes were nocturnal regardless of hunting effort or recreational activity intensity (Figure 5). Lynxes slightly shifted their activity toward daylight hours under medium hunting effort.

### Nocturnality model

While activity curves display the circadian rhythm of wildlife, nocturnality quantifies a specific element of wildlife activity, that is, the proportion of nighttime activity that has been shown to be greatly influenced by human activities (Gaynor et al., 2018). This warrants a closer inspection of the effects of hunting and recreational disturbance on wildlife nocturnality.

**FIGURE 6:**
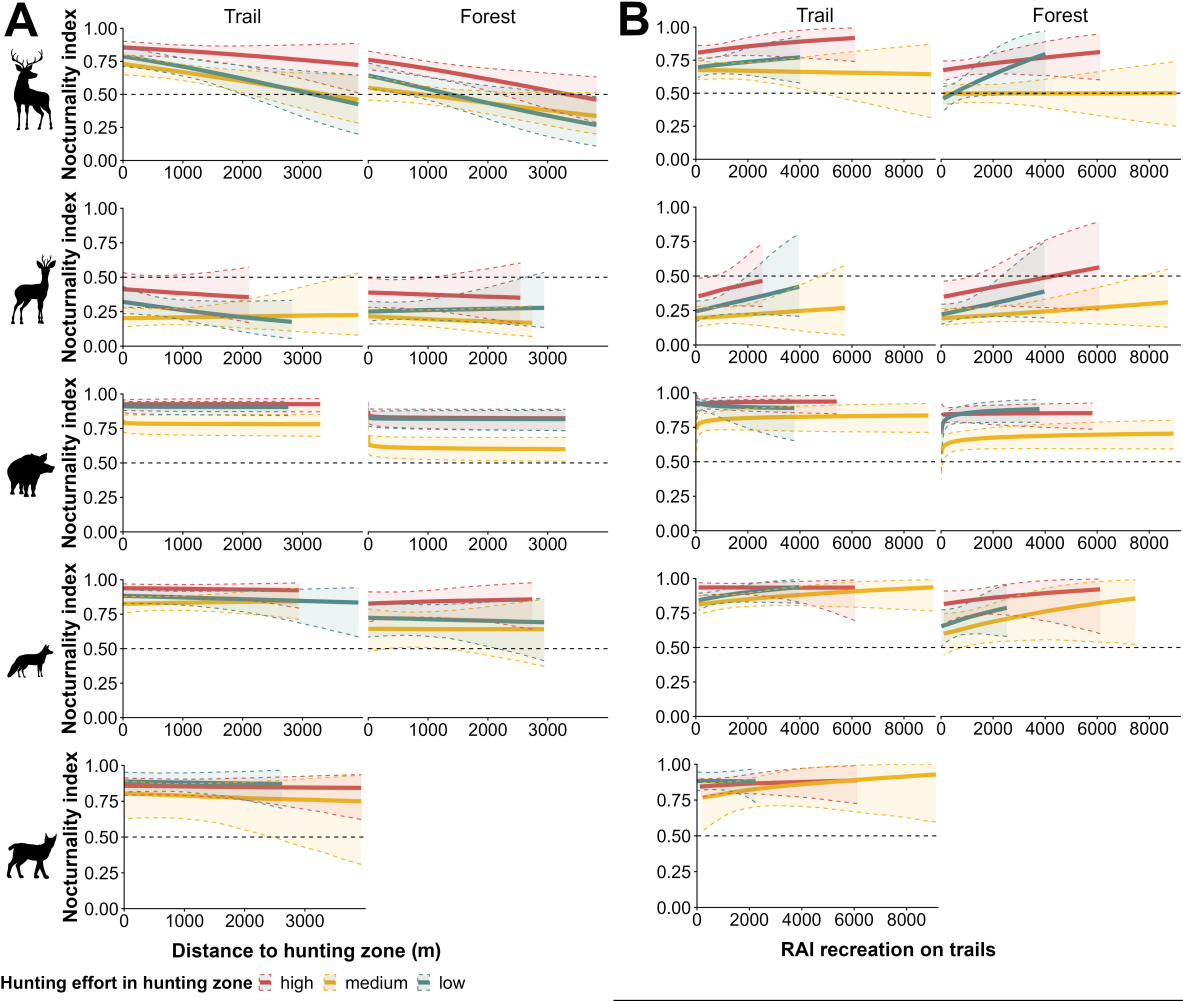
Effect of (A) distance to hunting zone (m) and (B) relative abundance index (RAI) of recreation activity on trails under each hunting effort (red line = high, yellow line = medium, blue line = low, dotted lines = credible intervals) on the nocturnality index of red deer, roe deer, wild boar, red fox and lynx (top to bottom). An index of 0.5 (dashed line) represents an equal activity level during night and day, while an index above 0.5 indicates nocturnal activity, and an index below 0.5 indicates diurnal activity. Animal silhouettes by Anne Peters.

Red deer were significantly more nocturnal on camera traps placed on trails (Appendix S1: Table S4). Their nocturnality decreased significantly with hunting effort and as the distance to the hunting zone increased. Red deer activity shifted from nocturnal to diurnal in forests with increasing distance to the hunting zone at low and medium hunting intensities (Figure 6A, Appendix S1: Table S4). Although recreational activity on trails did not significantly affect red deer nocturnality, there was a nonsignificant trend for red deer to become more nocturnal in forests at low hunting effort when recreational trail use increased (Figure 6A, Appendix S1: Table S4). At medium hunting effort, we found a nonsignificant trend of increasing red deer nocturnality at forest locations as visibility increased, that is, vegetation cover decreased (Appendix S1: Figure S7, Table S4).

Roe deer became more diurnal as hunting effort decreased, with a significant difference between medium and high hunting effort. Neither the distance to the hunting zone, recreational activity on trails, nor camera placement significantly impacted roe deer nocturnality. However, there was a nonsignificant trend of increasing nocturnality with increasing recreational activity on trails (Figure 6B, Appendix S1: Table S5).

For wild boar, neither the distance to the hunting zone nor recreational activity on trails affected nocturnality (Figure 6A,B, Appendix S1: Table S6). However, wild boar were significantly less nocturnal during medium hunting effort and in forests compared to trails. There was a nonsignificant trend for increasing nocturnality with increasing visibility at forest locations during medium hunting effort (Appendix S1: Table S6, Figure S7).

Red foxes were significantly less nocturnal in forests compared to trails and during low and medium hunting effort compared to high hunting effort (Figure 6A,B, Appendix S1: Table S7). Increasing recreational activity led to a nonsignificant trend of nocturnality increasing (Figure 6B, Appendix S1: Table S7), while distance to the hunting zone did not affect red fox nocturnality (Figure 6A, Appendix S1: Table S7).

Lynx remained predominantly nocturnal, showing no significant response to hunting effort, distance to the hunting zone, or recreational activity on the trails (Figure 6A,B, Appendix S1: Table S8). However, there was a nonsignificant trend for lynx to be less nocturnal during medium hunting effort with increasing visibility (Appendix S1: Figure S7, Table S8).

## DISCUSSION

Our analysis revealed a strong impact of varying hunting effort on wildlife nocturnality and spatial avoidance of areas connected to recreational activity, that is, trails. Red deer showed the most pronounced temporal and spatial adjustments to varying hunting effort. Wild boar and non‐hunted species showed moderate or negligible responses to variable hunting effort. The limited response of wild boar and lack of significant behavioral shifts in lynx indicate that other factors, such as specific species traits, may moderate these effects. Most species, except for roe deer, shifted their activity toward night on trails in response to recreational activity, regardless of hunting effort. Moreover, red deer, wild boar, and red fox were always significantly more nocturnal on trails compared to in forests. Our results indicate a consistent temporal avoidance by wildlife of areas where recreational activity frequently occurs, irrespective of hunting effort and intensity of recreational activity. However, spatial avoidance of trails decreased in ungulates as hunting effort declined, particularly in red deer. Varying intensity of recreational activity had only limited effects across all species, with only subtle trends, for example, a slight increase in nocturnality with increasing recreational trail use in red deer at forest locations during low hunting effort and for roe deer and red fox in general.

Boone et al. (2025) employed a similar camera trap design, positioning cameras on trails and 50 m away from trails. They showed that human activity primarily influenced wildlife behavior on trails, with minimal effects extending beyond 50 m. In contrast, our study detected behavioral changes at a 100 m distance from trails. This difference may stem from the fact that hunting was prohibited in their study area, suggesting that the combined effects of hunting effort and recreational activity influenced our results, especially for red deer. Red deer were predominantly nocturnal during high hunting effort, regardless of recreation intensity and at both trail and forest locations. However, they showed a more distinct response to varying recreation activity at forest locations when hunting effort was low, only temporally avoiding areas near trails with high recreational activity. Similarly, roe deer and red fox increased their temporal avoidance of recreational activity only during high hunting effort. In the case of our study, varying hunting effort coincided with the season. However, previous studies on the temporal activity of the target species found no or very limited changes regarding temporal activity patterns over the year that could be explained by natural variations in wildlife behavior (Ensing et al., 2014; Ikeda et al., 2016; Johann et al., 2020; Kamler, 2007; Keuling et al., 2008; Krop‐Benesch et al., 2013; Monterroso et al., 2014; Podolski et al., 2013). This supports the argument that also in the case of our study, human disturbances are a stronger driver of varying temporal activity than seasonality.

We hypothesized (H1) that the target species adjust their temporal activity in response to hunting effort and intensity of recreational activity. That is because hunting and recreational activities inside the BFNP are highly predictable, considering both space and time. Humans are active during the day and mostly on trails, while hunting is restricted to the hunting zone and usually conducted during twilight. If the risk is predictable, wildlife should learn to avoid certain areas only when a potential predator is present (Creel et al., 2005; Cromsigt et al., 2013; Gaynor et al., 2019). Accordingly, red deer displayed a flexible response considering their temporal activity, regarding both spatial and temporal variations in human disturbance, supporting H1. They became less nocturnal as hunting effort and recreational activity decreased, particularly at forest sites. When human‐related risks were very low (e.g., during periods of low hunting effort, in non‐hunting zones, and near trails with low recreational use), red deer were diurnal. A similar pattern was observed in Białowieża National Park, Poland, where hunting is banned year‐round, and human activity is minimal, leading to mostly diurnal behavior (Kamler, 2007). Conversely, red deer were consistently nocturnal under high hunting risk, even when recreational activity was low. This suggests hunting as a main driver of nocturnality, which amplifies avoidance of recreation. However, while red deer generally showed no response to changes in recreational intensity, they became more nocturnal in forests near trails with increasing recreational activity during low hunting effort. This implies that recreation also influences temporal activity in areas surrounding trails, as noted by Marion et al. (2021). While red deer responded strongest to varying recreational activity under low hunting risk, tolerating lower levels of recreation, they avoided even low levels of recreation as hunting effort increased. This effect was more pronounced in the hunting zone, further indicating an additive effect of hunting effort.

Roe deer and red fox showed limited responses to varying recreation intensity and hunting effort, partially supporting H1. Roe deer were primarily diurnal, with activity peaks in the morning and afternoon at both trail and forest locations. However, during high hunting effort, they shifted to being predominantly crepuscular. Recreational activity had minimal impact, though there was a slight tendency for roe deer to avoid higher levels of recreation. This seems to be more pronounced during high hunting effort according to activity plots, suggesting that hunting amplifies avoidance of recreation activity. Since roe deer hunting was banned in the BFNP in 2012, they have become more diurnal compared to earlier studies (Krop‐Benesch et al., 2013; Pagon et al., 2013). Roe deer in the BFNP are heavily predated by lynx, a nocturnal ambush predator (Filla et al., 2017; Heurich et al., 2012; Möst et al., 2015). To reduce predation risk, roe deer may have shifted their activity toward daytime activity. This suggestion is in line with the findings of Bonnot et al. (2020), who observed that roe deer became more diurnal in areas with high lynx densities and no hunting pressure. Moreover, roe deer may use time periods when humans are most active as a shield from predation because lynx generally avoid humans (Belotti et al., 2018; Berger, 2007; Filla et al., 2017). This behavior may indicate a trade‐off between avoiding human disturbance and lowering lynx predation risk.

Red foxes were predominantly nocturnal on the trails but significantly less so in the forest year‐round, where they were rather cathemeral, especially during medium and low hunting effort. This aligns with previous findings of temporal avoidance of areas connected to human activities (Díaz‐Ruiz et al., 2015; Haswell et al., 2020), and cathemeral behavior in areas with low human activity and restricted hunting (Ikeda et al., 2016). Their nocturnal activity on trails likely reduces the risk of human encounters, who are active on trails during the day. In contrast, human activity is low in forests, enabling red foxes to remain active throughout the day. However, in hunting zones during high hunting effort, they also avoided times of recreational activity in forests, suggesting an additive effect of hunting and recreation. As red fox observations at forest sites were, however, limited, additional data would be beneficial to verify this observation.

Red foxes and roe deer may react to high hunting effort inside the BFNP because both species experience hunting pressure outside the national park's borders. Hunted populations of roe deer and red fox displayed similar activity patterns to those observed when hunting effort was high (Bonnot et al., 2020; Kämmerle et al., 2020), suggesting that non‐target species can indeed be affected by higher hunting effort due to increasing disturbances. In line with this, Brown et al. (2023) found that bears reacted to disturbance by moose hunting, even if they were not specifically targeted.

Lynx remained nocturnal on trails year‐round and showed no response to changes in hunting pressure or recreational activity, contradicting H1. This consistent nocturnality aligns with previous findings in the BFNP (Podolski et al., 2013) and other study sites, where lynx temporal activity was unaffected by human activity (Blašković et al., 2022; Haswell et al., 2020; Smith et al., 2024). Lynx may also not respond to increased hunting disturbances due to them being protected from hunting outside the BFNP (apart from illegal killings, Heurich et al., 2018). Although roe deer, the lynx's main prey in the BFNP, shifted toward diurnal activity after roe deer hunting stopped, lynx did not adjust their activity (Podolski et al., 2013). While temporal alignment with prey activity has been observed elsewhere (Naderi et al., 2021), roe deer in the BFNP remain active around twilight to a certain extent, and lynx are well adapted to hunt during low‐light conditions (Podolski et al., 2013). Thus, a shift to daytime activity by lynx is likely unnecessary to maintain hunting success.

Wild boar showed little to no variation in response to varying hunting or recreation disturbances and remained nocturnal throughout the study, contradicting H1. Similar to red deer, wild boar regularly encounter humans as lethal predators in the BFNP. Therefore, they likely avoid daytime activity to minimize the risk of encountering potentially dangerous humans. This behavior is probably more pronounced in wild boar due to year‐round hunting. This contrasts somewhat with the results of Parsons et al. (2022), who found lower temporal avoidance and stronger spatial avoidance of humans in hunted species under year‐round hunting. However, wild boar in the BFNP might be unable to spatially avoid humans permanently without losing access to important resources due to the dense network of hiking trails. Therefore, sustained nocturnality might be a more sustainable response to avoid human encounters. Wild boar reduced nocturnality only during medium hunting effort, with some daylight activity in forests near trails with low recreational use. While previous studies also observed wild boar to be predominantly nocturnal, they also found similar patterns of increased diurnal activity during summer in areas with low recreational and hunting activity (Johann et al., 2020; Keuling et al., 2008; Reinke et al., 2021). In our case, this behavior is likely linked to dense vegetation providing refuge during medium hunting effort (June–September), indicated by a trend of lower nocturnality when visibility was low, that is, vegetation cover high. We found a similar effect of vegetation cover during medium hunting effort for red deer in forests and lynx on trails. These results are in line with previous studies, showing that vegetation cover can mitigate the effects of human disturbance in both ungulates (Jayakody et al., 2008; Lone et al., 2015; Zong et al., 2023a, 2023b) and carnivores (Brown et al., 2023; Ordiz et al., 2011; Versluijs et al., 2022).

We further hypothesized (H2) that hunting effort would influence wildlife behavior in the non‐hunting zone, diminishing with distance from the hunting zone. This was confirmed for red deer, who exhibited stronger diurnal behavior in the non‐hunting zone, and decreasing nocturnality with increasing distance from the hunting zone. However, no such effect was observed for wild boar, roe deer, red fox, or lynx, contradicting H2. For roe deer, red fox, and lynx, this lack of response might be because these species are not hunted in the BFNP and, therefore, do not experience reduced risk in the non‐hunting zone that would prompt a behavioral change. Wild boar, however, should benefit from a lower hunting risk in the hunting‐free zone but remained nocturnal even at the maximum distance possible considering the BFNP's zoning, that is, 4 km. Johann et al. (2020) predicted higher diurnal activity of wild boar in the non‐hunting zone compared to the neighboring hunting zone, except during the main hunting season. They argued that animals learn to assess risk based on experience in both zones and adjust their behavior accordingly. As wild boar likely do not restrict their space use to the non‐hunting zone of the BFNP, they probably learned from their experiences in the hunting zone to be cautious of humans throughout the area to minimize the risk of being killed. Likewise, when hunting effort in the hunting zone was high, red deer remained nocturnal up to several kilometers from the hunting zone. Our observation that hunting effort affects wildlife behavior several kilometers into non‐hunting zones indicates the importance of creating extensive hunting‐free areas to reduce the impact of hunting activities on wildlife efficiently. Unfortunately, establishing large hunting‐free zones covering the home ranges of large species is often not achieved in European national parks (Van Beeck Calkoen et al., 2020).

Our results confirm for red deer, roe deer, and wild boar that spatial avoidance of trails increased with higher hunting effort (H3). While we expected ungulates to be more frequently recorded on forest cameras, trails within the BFNP often intersect their home ranges. Therefore, we anticipated some ungulate presence on trails, for example, when crossing or feeding nearby. Consistent with this expectation, we observed higher RAIs in forests compared to on‐trail for all three species across all hunting efforts, except for red deer during low hunting effort. However, as hunting effort decreased, the trail index increased for all ungulates. This indicates that ungulates were observed with a higher relative frequency on trails when hunting effort was lower, suggesting that spatial avoidance of areas linked to recreational activity is influenced by hunting effort. In contrast, red foxes and lynxes were much more frequently recorded on trails, aligning with our predictions and with previous observations of carnivores using trails often to ease movement through their territories (Bandak et al., 2020; Kays et al., 2017). Their use of trails did not seem affected by hunting effort. However, red fox RAI and trail index increased during medium hunting effort, while lynxes showed a similar increase during high hunting effort. These patterns likely reflect periods during which juveniles of red fox (late summer) and lynx (autumn/winter) are starting to become more independent, leading to increased observations of these species (Sarmento et al., 2021; Weingarth et al., 2015).

Our findings only partially support the hypothesis (H4) that hunted species exhibit stronger responses to increased hunting effort. As predicted, red deer showed clear temporal shifts toward nocturnality and greater trail avoidance under high hunting effort. However, wild boar displayed limited changes in activity or trail avoidance, contradicting H4. We also only partially confirmed the hypothesis (H5) that non‐target species are affected by increasing hunting disturbances. Roe deer and red fox significantly adjusted their temporal activity, particularly during periods of high hunting effort, despite not being actively hunted in the BFNP. In contrast, lynx showed no behavioral changes in response to hunting effort. This may be due to their pronounced nocturnal behavior and their protected status, which shields them from direct hunting pressure.

Our study provides novel insights into the interactive effects of recreation and hunting on large mammal communities in protected areas, using detailed information on both activities. Fine‐scale information on anthropogenic disturbances is often missing when assessing the impacts of human activities on wildlife. Expanding our study framework to other regions across Europe would be beneficial to enhance our understanding of the combined impact of hunting and non‐lethal recreation on wildlife at a broader scale. Such datasets would be especially valuable for assessing the interactive effects of different intensities of recreation and hunting on the spatial behavior of wildlife, for example, habitat use and movement. Our analyses regarding lynx were limited by the low number of observations on forest cameras, which could be overcome by extending the study period to several years or deploying a denser network of camera traps to better monitor rare species. Since higher hunting effort also affected non‐target species, we suggest that more research should be conducted on the effect of hunting on non‐target species of conservation concern. This is especially relevant for conservation areas, which are often designated to protect disturbance‐sensitive species that can experience fitness costs due to increasing human disturbances.

From a conservation perspective, the temporal displacement of wildlife can affect species interactions, potentially impacting underlying ecosystem functions (Wilson et al., 2020). For instance, a shift in roe deer activity toward crepuscular/nocturnal activity during high hunting effort increases the temporal overlap with their predator, the lynx, potentially reducing roe deer survival (Gehr et al., 2018). Changes in herbivore behavior may have cascading effects on lower trophic levels, as herbivores often play an important role in seed dispersal and nutrient cycling (Kuijper et al., 2010; Murray et al., 2013; Wright et al., 2020). We understand that strictly prohibiting hunting in national parks is often not possible because hunting is considered an important management tool for reducing conflicts with neighboring landowners. However, managers must be aware of the strong impact of hunting on wildlife behavior. Spatially and temporally restricting hunting as far as possible would facilitate natural processes, better aligning protected areas with their management goals.

## AUTHOR CONTRIBUTIONS

Anne Peters, Adam F. Smith, and Marco Heurich conceptualized the study. The camera trap design was conceived by Anne Peters and Marco Heurich, and Anne Peters collected and processed the data. Analyses were conducted by Anne Peters with main contributions from Adam F. Smith and support by Maik Henrich and Carsten F. Dormann. Anne Peters led the writing, and all authors helped with editing and revising the manuscript and approved the publication of this paper.

## CONFLICT OF INTEREST STATEMENT

The authors declare no conflicts of interest.

## Supporting information


Appendix S1.

## Data Availability

Data (Peters et al., 2025) are available in Mendeley Data at https://doi.org/10.17632/pwz8b5wnpw.1.
