# Supplementary material for: Temporal displacement of the mammal community in a protected area due to hunting and recreational activities
Source: Ecol Appl. 2025 Nov 2;35(7):e70118. doi: 10.1002/eap.70118 (PMC12580470; doi:10.1002/eap.70118)
Supplement: Supplementary file 1 — Appendix S1. [file EAP-35-e70118-s001.pdf]

Temporal displacement of the mammal community in a protected area due to hunting and recreational activities

Anne Peters, Adam F. Smith, Maik Henrich, Carsten F. Dormann, Marco Heurich

Ecological Applications

Appendix S1

Table S1 Criteria for trail and forest camera placement. Cameras were placed at least 200 meters from crossings to clearly assign each camera pair to a specific trail segment. A distance of 150 meters from houses and huts was maintained to avoid areas with high human activity unrelated to recreational use in the national park. The trail network within the national park's animal enclosures, which house native wildlife and are freely accessible to visitors, was excluded. Since this area is primarily visited by those observing wildlife, it is not relevant to the recreational activities analyzed in this study, i.e. hiking and biking.

**Criteria trail camera**	**Criteria forest camera**
At least 150 m distance to houses and huts	At least 150 m distance to houses and huts
Not within the animal enclosures' trail network	Not within the animal enclosures' trail network
At least 1 km distance to the next trail camera location	At least 100 m distance to all surrounding trails
At least 200 m from crossings/parking lots	Within the same habitat as the trail camera

Table S2 Information on trapping days per camera (Camera ID Forest/Trail) during each hunting effort (low, medium, high). Mean, median, minimum and maximum trapping days of camera traps for each hunting effort are stated at the bottom of the table.

Camer ID Forest	trapping days low hunting	trapping days medium hunting	trapping days high hunting	Camera ID Trail	trapping days low hunting	trapping days medium hunting	trapping days high hunting
F27	120	122	90	T27	68	69	109
F28	120	91	112	T28	117	120	116
F29	113	122	124	T29	120	122	124
F30	120	122	146	T30	120	122	146
F31	60	70	69	T31	120	122	125
F32	28	84	18	T32	120	62	125
F33	118	122	120	T33	120	114	103
F34	120	122	127	T34	120	101	129
F35	120	122	123	T35	120	122	123
F36	120	68	130	T36	120	122	127
F37	91	122	50	T37	100	113	107
F38	44	101	55	T38	120	122	128
F39	120	122	115	T39	120	122	124
F40	120	122	145	T40	120	122	145
F41	120	122	139	T41	120	122	136
F42	100	122	104	T42	120	122	123
F43	107	122	102	T43	120	122	115
F44	120	122	131	T44	120	122	126
F45	120	122	122	T45	120	122	122
F46	120	122	119	T46	120	122	63
F47	120	122	97	T47	120	122	127
F48	117	122	117	T48	120	122	63
F49	118	122	107	T49	67	122	117
F50	64	0	77	T50	73	0	82
F51	35	122	102	T51	120	122	128
F52	120	122	112	T52	44	108	40
F53	120	122	134	T53	120	98	141
F54	120	122	98	T54	89	122	129
F55	118	122	112	T55	120	122	124
F56	97	122	110	T56	120	122	130
F57	48	122	93	T57	111	122	92
F58	120	122	124	T58	120	122	124
F59	120	122	132	T59	120	122	132
F60	120	122	138	T60	120	122	145
F61	120	122	119	T61	110	122	109
F62	120	122	84	T62	120	122	82
F63	103	122	125	T63	120	121	146
F64	97	122	112	T64	120	122	129
F65	120	122	132	T65	120	122	138
F66	120	122	104	T66	120	122	138
F67	120	76	121	T67	120	122	121
F68	52	107	80	T68	84	122	112
F69	116	122	89	T69	53	122	104
F70	116	122	120	T70	120	103	115
F71	120	122	116	T71	120	122	124
F72	75	122	105	T72	120	122	125
F73	120	122	134	T73	120	122	134
F74	110	122	108	T74	120	122	123
F75	120	122	121	T75	91	122	126
F76	120	75	114	T76	120	122	109
F77	79	122	92	T77	108	122	117
F78	119	122	140	T78	120	60	108
F79	119	122	114	T79	120	122	140
F80	120	122	105	T80	120	122	79
F81	101	122	112	T81	120	122	117
F82	120	122	103	T82	120	122	125
F83	120	122	127	T83	120	122	127
F84	120	122	117	T84	120	122	118
F85	120	122	80	T85	120	55	74
F86	120	122	102	T86	119	122	117
F87	48	122	56	T87	48	122	56
**Mean**	106.11	115.02	108.62	**Mean**	111.51	114.43	116.44
**Median**	120	122	112	**Median**	120	122	124
**Minimum**	28	0	18	**Minimum**	44	0	40
**Maximum**	120	122	146	**Maximum**	120	122	146

Table S3 Specification of nocturnality models build for red deer, roe deer, wild boat, red fox and lynx. The models included a three-way interaction between camera placement (trail/forest), hunting effort (high, medium, low), and other variables: the relative abundance index (RAI) of recreational activity on trails, visibility (i.e. vegetation cover), and distance to the hunting zone. This allowed us to evaluate how the nocturnality index of each species varied between camera placement and hunting effort in response to these variables. For lynx, forest cameras were excluded due to the low number of observations at forest locations.

Species	Model specification
Red deer	RAI Recreation on trails + Visibility 140cm +<br>Distance to hunting zone [m] + Hunting effort + Camera placement +<br>RAI Recreation on trails : Hunting effort : Camera placement +<br>Distance to hunting zone [m] : Hunting effort : Camera placement +<br>Visibility 140cm : Hunting effort : Camera placement
Roe deer	RAI Recreation on trails + Visibility 140cm +<br>Distance to hunting zone [m] + Hunting effort + Camera placement +<br>RAI Recreation on trails : Hunting effort : Camera placement +<br>Distance to hunting zone [m] : Hunting effort : Camera placement +<br>Visibility 140cm : Hunting effort : Camera placement
Wild boar	RAI Recreation on trails (log) + (Visibility 70cm)^2 +<br>Distance to hunting zone [m] (log) + Hunting effort + Camera placement +<br>RAI Recreation on trails (log) : Hunting effort : Camera placement +<br>Distance to hunting zone [m] (log) : Hunting effort : Camera placement +<br>(Visibility 70cm)^2: Hunting effort : Camera placement
Red fox	RAI Recreation on trails + (Visibility 50cm)^2 +<br>Distance to hunting zone [m] + Hunting effort + Camera placement +<br>RAI Recreation on trails : Hunting effort : Camera placement +<br>Distance to hunting zone [m] : Hunting effort : Camera placement +<br>Visibility 50cm : Hunting effort : Camera placement
Eurasian lynx	RAI Recreation on trails + Visibility 70cm +<br>Distance to huntin g zone [m] + Hunting effort +<br>RAI Recreation on trails : Hunting effort +<br>Distance to hunting zone [m] : Hunting effort +<br>Visibility 70cm : Hunting effort

Table S4 Nocturnality model results for red deer, showing the estimate and 95% credible interval (CRI 95%) for each predictor variable. Italic and bold: significant predictors. Italic = predictors with CRI only slightly overlapping of zero, indicating a slight effect of this variable on the species' nocturnality. "High hunting effort" served as the reference level for hunting effort, and "forest" as the reference levels for camera placement.

**RED DEER**		
**Predictors**	**Estimates**	**CI (95%)**
Intercept	0.88	0.58 – 1.16
RAI recreation on trails (Recreation)	0.07	-0.11 – 0.44
Visibility [50cm]^2 (Visibility)	0.01	-0.18 – 0.20
Distance to hunting zone [m] (DHZ)	***-0.29***	***-0.51 – -0.04***
Hunting effort (HE) [low]	***-0.65***	***-1.06 – -0.20***
Hunting effort (HE) [medium]	***-0.86***	***-1.23 – -0.49***
Camera placement (CP) [trail]	***0.73***	***0.42 – 1.04***
DHZ : HE [high] : CP [forest]	-0.04	-0.40 – 0.20
DHZ : HE [low] : CP [forest]	-0.10	-0.56 – 0.13
DHZ : HE [medium] : CP [forest]	0.03	-0.19 – 0.36
DHZ : HE [high] : CP [trail]	0.05	-0.20 – 0.47
DHZ : HE [low] : CP [trail]	-0.10	-0.54 – 0.15
DHZ : HE [medium] : CP [trail]	-0.00	-0.30 – 0.28
Recreation : HE [high] : CP [forest]	0.06	-0.22 – 0.50
Recreation : HE [low] : CP [forest]	*0.46*	*-0.08 – 1.42*
Recreation : HE [medium] : CP [forest]	-0.06	-0.48 – 0.13
Recreation : HE [high] : CP [trail]	0.12	-0.21 – 0.89
Recreation : HE [low] : CP [trail]	0.03	-0.33 – 0.62
Recreation : HE [medium] : CP [trail]	-0.08	-0.53 – 0.13
Visibility : HE [high] : CP [forest]	0.12	-0.11 – 0.51
Visibility : HE [low] : CP [forest]	0.01	-0.23 – 0.34
Visibility : HE [medium] : CP [forest]	*0.14*	*-0.09 – 0.50*
Visibility : HE [high] : CP [trail]	-0.11	-0.55 – 0.14
Visibility : HE [low] : CP [trail]	-0.01	-0.35 – 0.21
Visibility : HE [medium] : CP [trail]	-0.11	-0.51 – 0.12
Observations	299	

Table S5. Nocturnality model results for roe deer, showing the estimate and 95% credible interval (CRI 95%) for each predictor variable. Italic and bold: significant predictors. Italic = predictors with CRI only slightly overlapping of zero, indicating a slight effect of this variable on the species' nocturnality. "High hunting effort" served as the reference level for hunting effort, and "forest" as the reference levels for Camera placement.

**ROE DEER**		
**Predictors**	**Estimates**	**CI (95%)**
Intercept	-0.52	-1.04 – -0.03
RAI recreation on trails (Recreation)	*0.12*	*-0.05 – 0.49*
Visibility [50cm]^2 (Visibility)	0.01	-0.15 – 0.24
Distance to hunting zone [m] (DHZ)	-0.03	-0.34 – 0.16
Hunting effort (HE) [low]	*-0.54*	*-1.13 – 0.01*
Hunting effort (HE) [medium]	***-0.89***	***-1.50 – -0.13***
Camera placement (CP) [trail]	0.03	-0.18 – 0.36
DHZ : HE [high] : CP [forest]	-0.00	-0.41 – 0.40
DHZ : HE [low] : CP [forest]	0.06	-0.19 – 0.60
DHZ : HE [medium] : CP [forest]	-0.03	-0.49 – 0.22
DHZ : HE [high] : CP [trail]	-0.02	-0.59 – 0.35
DHZ : HE [low] : CP [trail]	-0.20	-0.85 – 0.10
DHZ : HE [medium] : CP [trail]	0.05	-0.22 – 0.56
Recreation : HE [high] : CP [forest]	0.02	-0.26 – 0.46
Recreation : HE [low] : CP [forest]	0.08	-0.20 – 0.73
Recreation : HE [medium] : CP [forest]	-0.03	-0.40 – 0.16
Recreation : HE [high] : CP [trail]	0.04	-0.27 – 0.86
Recreation : HE [low] : CP [trail]	0.08	-0.20 – 0.79
Recreation : HE [medium] : CP [trail]	-0.02	-0.52 – 0.24
Visibility : HE [high] : CP [forest]	0.04	-0.23 – 0.52
Visibility : HE [low] : CP [forest]	0.00	-0.30 – 0.36
Visibility : HE [medium] : CP [forest]	0.01	-0.26 – 0.36
Visibility : HE [high] : CP [trail]	0.00	-0.36 – 0.43
Visibility : HE [low] : CP [trail]	-0.04	-0.44 – 0.21
Visibility : HE [medium] : CP [trail]	0.00	-0.31 – 0.32
Observations	203	

Table S6. Nocturnality model results for wild boar, showing the estimate and 95% credible interval (CRI 95%) for each predictor variable. Italic and bold: significant predictors. Italic = predictors with CRI only slightly overlapping of zero, indicating a slight effect of this variable on the species' nocturnality. "High hunting effort" served as the reference level for hunting effort, and "forest" as the reference levels for Camera placement.

**WILD BOAR**		
**Predictors**	**Estimates**	**CI (95%)**
Intercept	1.70	1.32 – 2.13
RAI recreation on trails (Recreation)	0.07	-0.13 – 0.34
Visibility [50cm]^2 (Visibility)	0.15	-0.10 – 0.49
Distance to hunting zone [m] (DHZ)	-0.07	-0.36 – 0.12
Hunting effort (HE) [low]	-0.10	-0.65 – 0.19
Hunting effort (HE) [medium]	***-1.14***	***-1.65 – -0.68***
Camera placement (CP) [trail]	***0.79***	***0.36 – 1.24***
DHZ : HE [high] : CP [forest]	-0.06	-0.58 – 0.23
DHZ : HE [low] : CP [forest]	0.00	-0.34 – 0.33
DHZ : HE [medium] : CP [forest]	-0.08	-0.51 – 0.17
DHZ : HE [high] : CP [trail]	0.07	-0.30 – 0.76
DHZ : HE [low] : CP [trail]	-0.00	-0.46 – 0.41
DHZ : HE [medium] : CP [trail]	-0.00	-0.39 – 0.38
Recreation : HE [high] : CP [forest]	-0.02	-0.45 – 0.29
Recreation : HE [low] : CP [forest]	0.20	-0.12 – 0.79
Recreation : HE [medium] : CP [forest]	0.04	-0.20 – 0.37
Recreation : HE [high] : CP [trail]	0.01	-0.48 – 0.66
Recreation : HE [low] : CP [trail]	-0.22	-1.22 – 0.17
Recreation : HE [medium] : CP [trail]	0.03	-0.24 – 0.46
Visibility : HE [high] : CP [forest]	-0.01	-0.50 – 0.36
Visibility : HE [low] : CP [forest]	0.18	-0.13 – 0.67
Visibility : HE [medium] : CP [forest]	*0.34*	*-0.03 – 0.79*
Visibility : HE [high] : CP [trail]	0.01	-0.45 – 0.60
Visibility : HE [low] : CP [trail]	-0.01	-0.48 – 0.33
Visibility : HE [medium] : CP [trail]	-0.10	-0.66 – 0.18
Observations	234	

Table S7. Nocturnality model results for red fox, showing the estimate and 95% credible interval (CRI 95%) for each predictor variable. Italic and bold: significant predictors. Italic = predictors with CRI only slightly overlapping of zero, indicating a slight effect of this variable on the species' nocturnality. "High hunting effort" served as the reference level for hunting effort, and "forest" as the reference levels for Camera placement.

**RED FOX**		
**Predictors**	**Estimates**	**CRI (95%)**
Intercept	1.66	0.99 – 2.42
RAI recreation on trails (Recreation)	*0.18*	*-0.08 – 0.65*
Visibility [50cm]^2 (Visibility)	0.00	-0.26 – 0.24
Distance to hunting zone [m] (DHZ)	-0.01	-0.29 – 0.25
Hunting effort (HE) [low]	***-0.74***	***-1.45 – -0.01***
Hunting effort (HE) [medium]	***-1.07***	***-1.80 – -0.33***
Camera placement (CP) [trail]	***1.01***	***0.42 – 1.55***
DHZ : HE [high] : CP [forest]	0.06	-0.40 – 0.98
DHZ : HE [low] : CP [forest]	-0.01	-0.51 – 0.38
DHZ : HE [medium] : CP [forest]	0.01	-0.46 – 0.52
DHZ : HE [high] : CP [trail]	-0.03	-0.56 – 0.35
DHZ : HE [low] : CP [trail]	-0.05	-0.54 – 0.21
DHZ : HE [medium] : CP [trail]	0.04	-0.26 – 0.51
Recreation : HE [high] : CP [forest]	0.02	-0.52 – 0.92
Recreation : HE [low] : CP [forest]	0.12	-0.37 – 1.38
Recreation : HE [medium] : CP [forest]	0.05	-0.32 – 0.70
Recreation : HE [high] : CP [trail]	-0.15	-0.93 – 0.22
Recreation : HE [low] : CP [trail]	0.15	-0.26 – 1.10
Recreation : HE [medium] : CP [trail]	0.01	-0.41 – 0.42
Visibility : HE [high] : CP [forest]	0.00	-0.56 – 0.61
Visibility : HE [low] : CP [forest]	-0.06	-0.56 – 0.28
Visibility : HE [medium] : CP [forest]	-0.04	-0.68 – 0.38
Visibility : HE [high] : CP [trail]	-0.02	-0.54 – 0.33
Visibility : HE [low] : CP [trail]	0.05	-0.19 – 0.49
Visibility : HE [medium] : CP [trail]	0.05	-0.19 – 0.48
Observations	226	

Table S8. Nocturnality model results for lynx, showing the estimate and 95% credible interval (CRI 95%) for each predictor variable. Italic and bold: significant predictors. Italic = predictors with CRI only slightly overlapping of zero, indicating a slight effect of this variable on the species' nocturnality. "High hunting effort" served as the reference level for hunting effort.

**EURASIAN LYNX**		
**Predictors**	**Estimates**	**CRI (95%)**
Intercept	1.77	1.35 – 2.26
RAI recreation on trails (Recreation)	0.07	-0.19 – 0.70
Visibility [70cm] (Visibility)	0.00	-0.30 – 0.29
Distance to hunting zone (log) [m] (DHZ)	-0.02	-0.38 – 0.25
Hunting effort (HE) [low]	0.17	-0.18 – 1.18
Hunting effort (HE) [medium]	-0.41	-1.45 – 0.09
DHZ : HE [low]	-0.00	-0.56 – 0.50
DHZ : HE [medium]	-0.02	-0.61 – 0.41
Recreation: HE [low]	-0.04	-1.07 – 0.52
Recreation: HE [medium]	0.05	-0.29 – 0.90
Recreation: HE [low]	0.00	-0.41 – 0.46
Visibility : HE [medium]	*0.47*	*-0.06 – 1.51*
Observations	104	

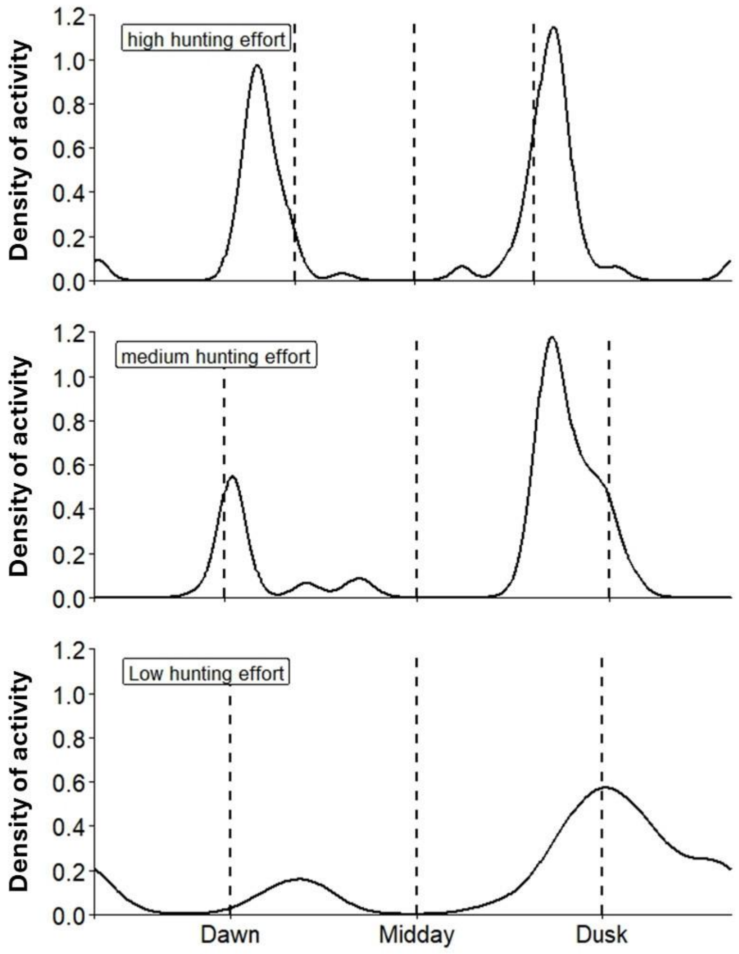

Fig. S1 Activity density plots of hunting events during high (top row), medium (middle row), and low (bottom row) hunting effort. The dashed vertical lines indicate dawn, midday, and dusk (from left to right).

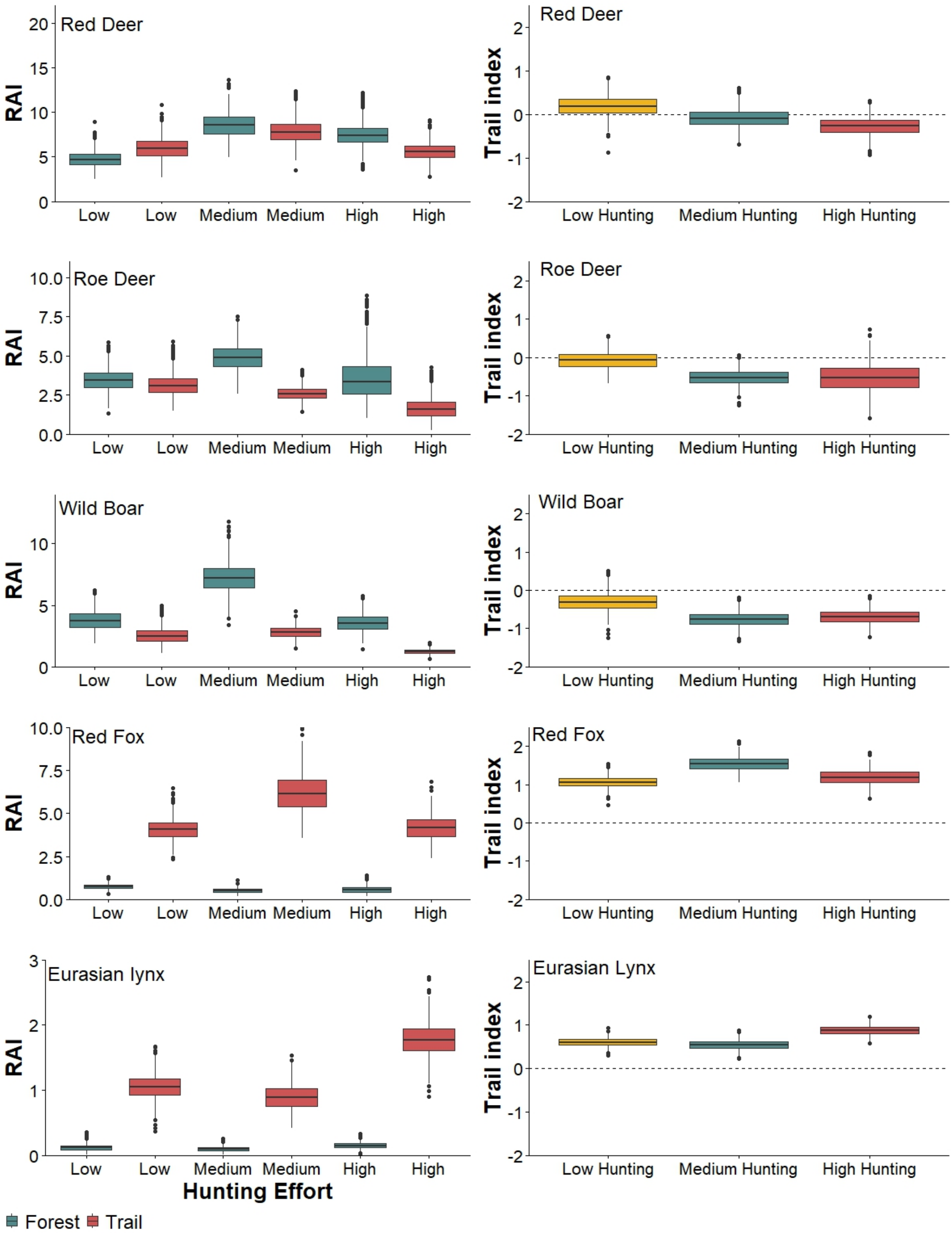

Fig. S2 Left panels: Boxplots show bootstrapped Relative Abundance Indices (RAI) for red deer, roe deer, wild boar, red fox, and lynx (from top to bottom) based on data from forest cameras (blue) and trail cameras (red). Right panels: Boxplots display bootstrapped trail index values for red deer, roe deer, wild boar, red fox, and lynx (from top to bottom) during periods of low (yellow), medium (blue), and high (red) hunting effort. The horizontal dashed line at a trail index of 0 indicates no preference between trail and forest locations. A trail index above 0 suggests the species was recorded more frequently on trails, while a value below 0 indicates more frequent recordings in forested areas.

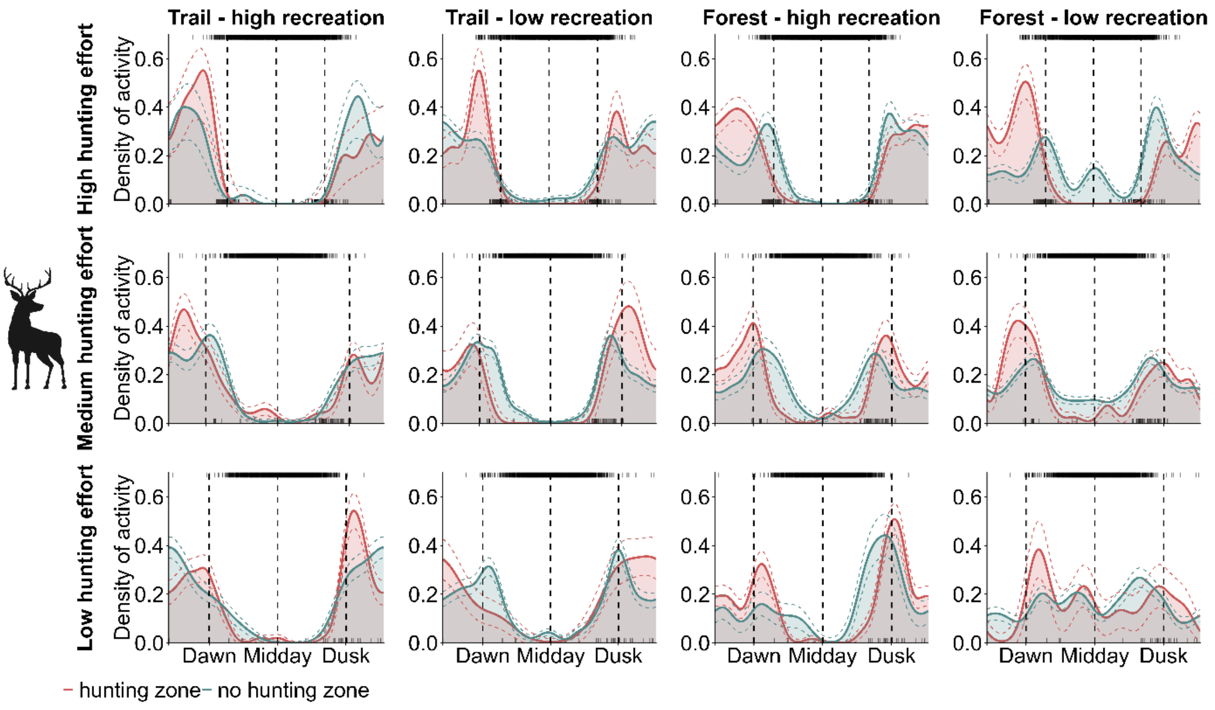

Fig. S3 Activity density curves for red deer. The red curves represent activity density curves in the hunting zone, while the blue curves represent activity density curves in the non-hunting zone. Dotted lines indicate confidence intervals. The plots are organized by hunting effort, with high effort shown in the top row, medium in the middle row, and low in the bottom row. The columns, from left to right, show results for cameras on trails with high recreational activity, cameras on trails with low recreational activity, forest cameras near trails with high recreational activity, and forest cameras near trails with low recreational activity. The top rug in each plot shows the temporal distribution and density of recreational activity on trails, while the bottom rug shows the temporal distribution and density of hunting events. Dashed vertical lines mark dawn (left), midday (middle), and dusk (right), based on the sun's position. Missing curves indicate there were not enough independent observations (n observations < 10) to produce reliable activity estimates. ©animal silhouettes: Anne Peters

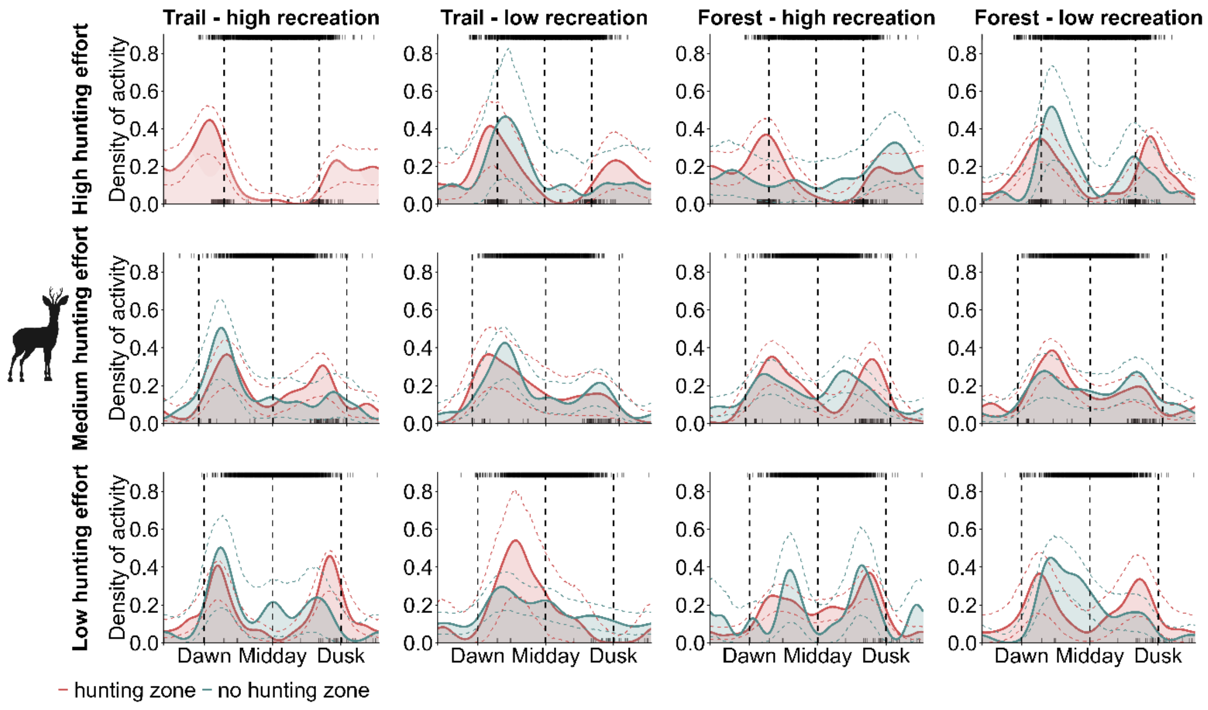

Fig. S4 Activity density plots for roe deer. The red curves represent activity density curves in the hunting zone, while the blue curves represent activity density curves in the non-hunting zone. Dotted lines indicate confidence intervals. The plots are organized by hunting effort, with high effort shown in the top row, medium in the middle row, and low in the bottom row. The columns, from left to right, show results for cameras on trails with high recreational activity, cameras on trails with low recreational activity, forest cameras near trails with high recreational activity, and forest cameras near trails with low recreational activity. The top rug in each plot shows the temporal distribution and density of recreational activity on trails, while the bottom rug shows the temporal distribution and density of hunting events. Dashed vertical lines mark dawn (left), midday (middle), and dusk (right), based on the sun's position. Missing curves indicate there were not enough independent observations (n observations < 10) to produce reliable activity estimates. ©animal silhouettes: Anne Peters

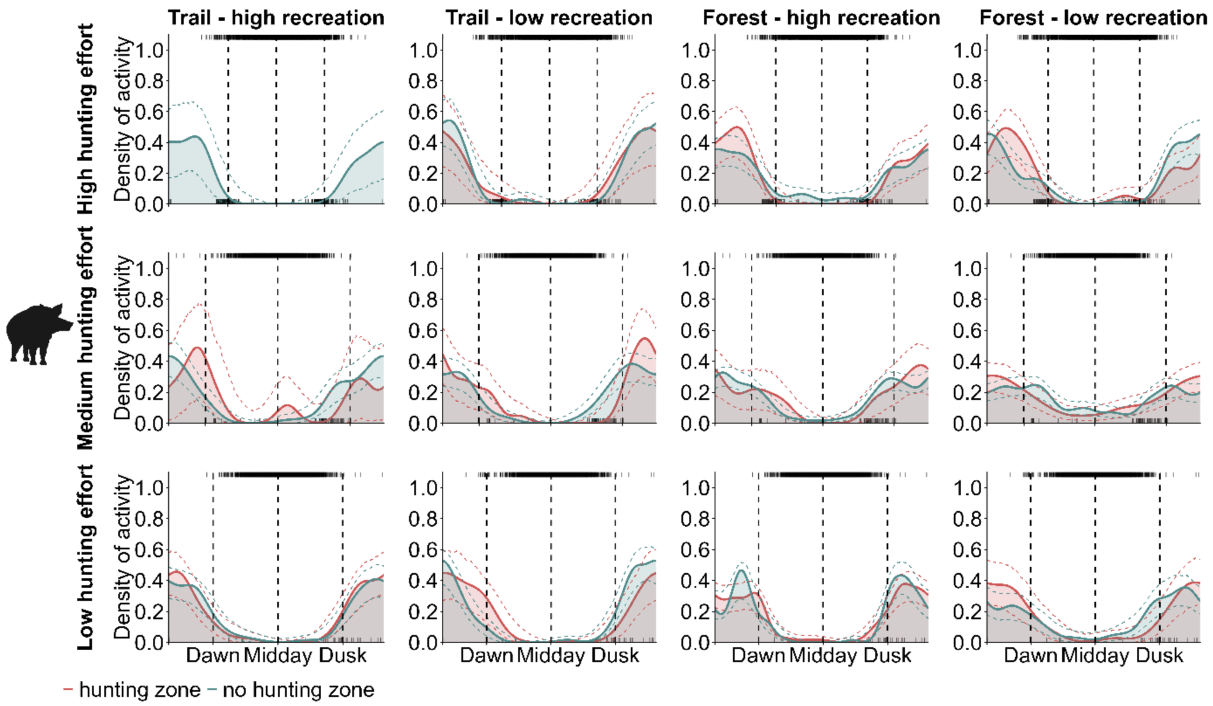

Fig. S5 Activity density plots for wild boar. The red curves represent activity density curves in the hunting zone, while the blue curves represent activity density curves in the non-hunting zone. Dotted lines indicate confidence intervals. The plots are organized by hunting effort, with high effort shown in the top row, medium in the middle row, and low in the bottom row. The columns, from left to right, show results for cameras on trails with high recreational activity, cameras on trails with low recreational activity, forest cameras near trails with high recreational activity, and forest cameras near trails with low recreational activity. The top rug in each plot shows the temporal distribution and density of recreational activity on trails, while the bottom rug shows the temporal distribution and density of hunting events. Dashed vertical lines mark dawn (left), midday (middle), and dusk (right), based on the sun's position. Missing curves indicate there were not enough independent observations (n observations < 10) to produce reliable activity estimates. ©animal silhouettes: Anne Peters

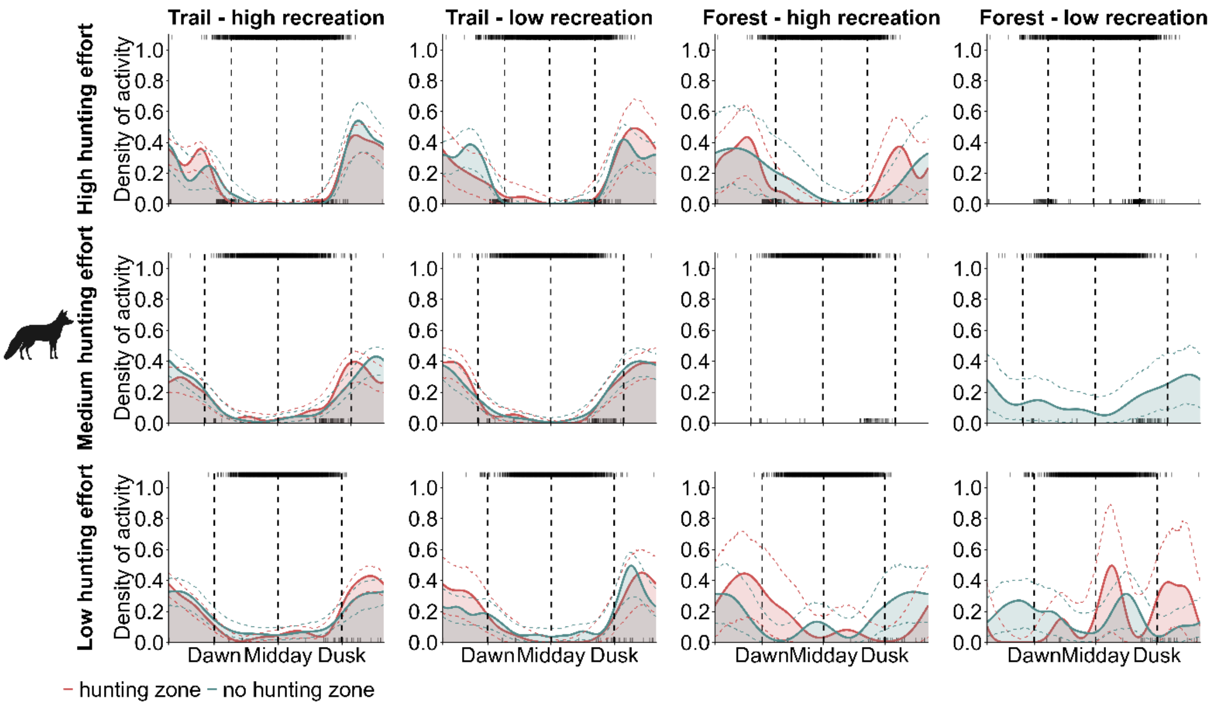

Fig. S6 Activity density plots for red fox. The red curves represent activity density curves in the hunting zone, while the blue curves represent activity density curves in the non-hunting zone. Dotted lines indicate confidence intervals. The plots are organized by hunting effort, with high effort shown in the top row, medium in the middle row, and low in the bottom row. The columns, from left to right, show results for cameras on trails with high recreational activity, cameras on trails with low recreational activity, forest cameras near trails with high recreational activity, and forest cameras near trails with low recreational activity. The top rug in each plot shows the temporal distribution and density of recreational activity on trails, while the bottom rug shows the temporal distribution and density of hunting events. Dashed vertical lines mark dawn (left), midday (middle), and dusk (right), based on the sun's position. Missing curves indicate there were not enough independent observations (n observations < 10) to produce reliable activity estimates. ©animal silhouettes: Anne Peters

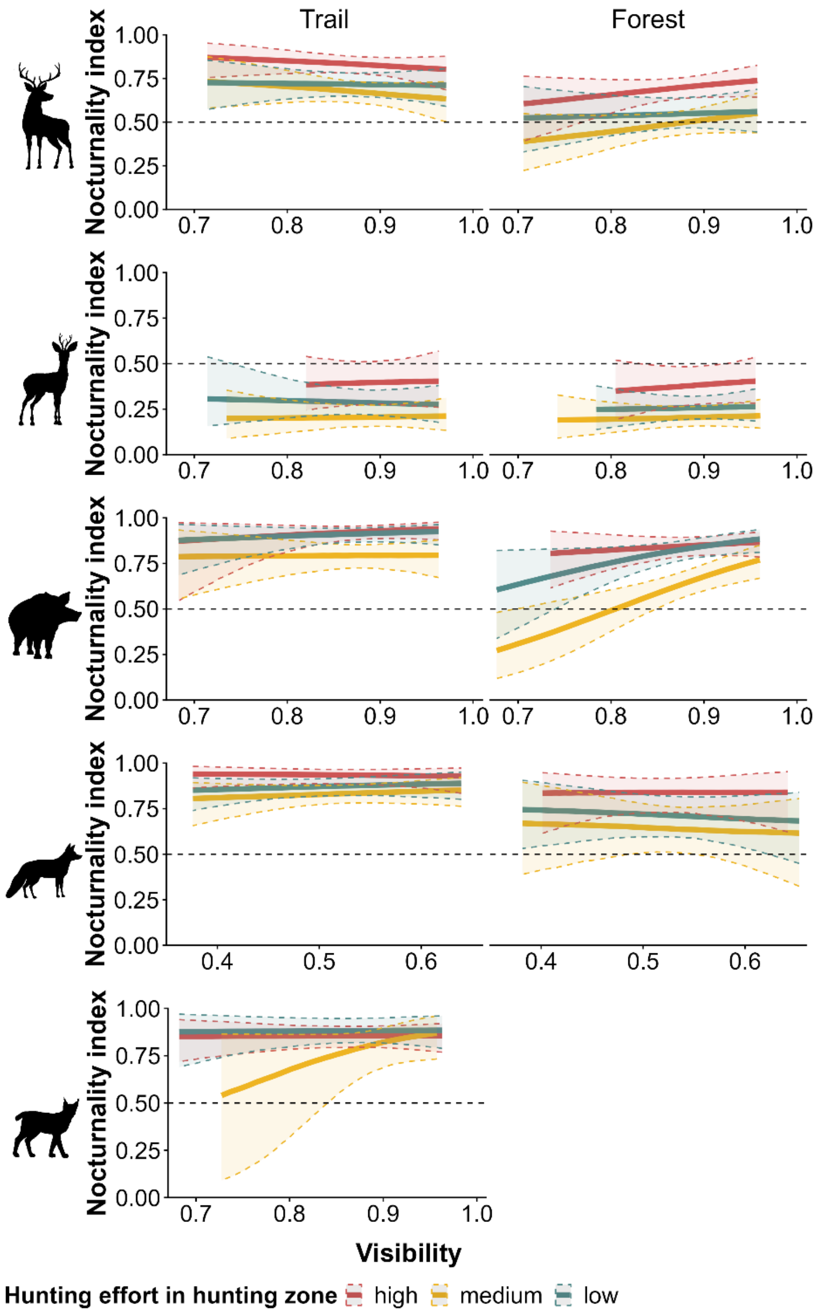

Fig. S7 Effect of increasing visibility under varying hunting effort (red line = high, yellow line = medium, blue line = low, dotted lines = credible intervals) on the nocturnality index of red deer, roe deer, wild boar, red fox and lynx (top to bottom). An index of 0.5 (dashed line) represents an equal activity level during night and day, while an index above 0.5 indicates nocturnal activity and an index below 0.5 indicates diurnal activity. ©animal silhouettes: Anne Peters
